# Impact of limited residential address on health effect analysis of predicted air pollution in a simulation study

**DOI:** 10.1038/s41370-022-00412-1

**Published:** 2022-01-26

**Authors:** Yoon-Bae Jun, Insang Song, Ok-Jin Kim, Sun-Young Kim

**Affiliations:** 1 Department of Statistics, Iowa State University, 1212, Snedecor Hall, 2438 Osborn Dr, Ames, IA 50011 USA; 2 Department of Geography, University of Oregon, Eugene, OR 97403 USA; 3 Environmental Health Research Department, Environmental Health Research Division, National Institute of Environmental Research, Incheon, Korea; 4 Department of Cancer Control and Population Health, Graduate School of Cancer Science and Policy, National Cancer Center, Goyang-si, Gyeonggi-do Korea

**Keywords:** Address, Exposure prediction, Health effect, Long-term exposure, Particulate matter

## Abstract

**Background:**

Recent epidemiological studies of air pollution have adopted spatially-resolved prediction models to estimate air pollution concentrations at people’s homes. However, the benefit of these models was limited in many studies that used existing health data relying on incomplete addresses resulting from confidentiality concerns or lack of interest when designed.

**Objective:**

This simulation study aimed to understand the impact of incomplete addresses on health effect estimation based on the association between particulate matter with diameter ≤10 µm (PM_10_) and low birth weight (LBW).

**Methods:**

We generated true annual average concentrations of PM_10_ at 46,007 mothers’ homes and their LBW status, using the parameters obtained from our data analysis and a previous study in Seoul, Korea. Then, we hypothesized that mothers’ address information is limited to the district and compared the properties of their health effect estimates of PM_10_ with those using complete addresses. We performed this comparison across eight environmental scenarios that represent various spatial distributions of PM_10_ and nine exposure prediction methods that provide different sets of predicted PM_10_ concentrations of mothers.

**Results:**

We observed increased bias and root mean square error consistently across all environmental scenarios and prediction methods using incomplete addresses compared to complete addresses. However, the bias related to incomplete addresses decreased when we used population-representative exposures averaged to the district from predicted PM_10_ at census tract centroids.

**Significance:**

Our simulation study suggested that individual exposure estimated by prediction approaches and averaged across population-representative points can provide improved accuracy in health effect estimates when complete address data are unavailable.

**Impact statement:**

Our simulation study focused on a common and practical challenge of limited address information in air pollution epidemiology, and investigated its impact on health effect analysis. Cohort studies of air pollution have developed advanced exposure prediction model to allow the estimation of individual-level long-term air pollution concentrations at people’s addresses. However, it is common that address information of existing health data is available at the coarse spatial scale such as city, district, and zip code area. Our findings can help understand the possible consequences of limited address information and provide practical guidance in achieving the accuracy in health effect analysis.

## Introduction

Long-term exposure to air pollution was associated with mortality and morbidity in many epidemiological studies and the investigation was expanded to large health data such as multi-city or multi-country cohorts [1–4]. Accurate assessment of individual exposure to long-term air pollution has been crucial in these studies, as individual air pollution measurements are not available given financial and technical constraints. Recent studies developed exposure prediction models to represent substantial spatial variability of exposures across study participants and enhanced the capacity to assess the association with human health. These models produced air pollution concentrations estimated at people’s homes or workplaces as their individual-level exposures [5, 6]. Specifically, physicochemical models relied on emissions and meteorology data and estimated air pollution concentrations on the grid [7, 8]. Statistical models were mostly constructed in pointwise regression including geographical and/or meteorological characteristics, named land use regression [9–11]. Additional spatial correlation structure was modelled by using geostatistical techniques such as kriging [12, 13] and spatial smoothing as applied in generalized additive model [14].

The benefit of these advanced exposure prediction approaches could be limited when complete address data are unavailable. It is common that address information of existing health data is available at the coarse spatial scale such as city, district, and zip code [15–22]. Existing cohorts were often not designed to collect full address data [1, 23–26]. This limitation is more common in administrative health data constructed based on census or public health insurance. Despite their strength of large representative populations that allow examining the association at the national or regional scale, address data were restricted given the concerns of confidentiality [16–24, 27]. For instance, studies using the U.S. Medicare cohort, the Canadian Census cohort, and the Taiwan National Health Insurance Database cohort assessed individual exposure to air pollution at the zip code or postal code area which is the finest spatial resolution of available address data [17, 19, 21, 22]. This incomplete address data may increase exposure misclassification and affect the accuracy and/or precision of health effect estimates.

This simulation study aimed to understand the impact of incomplete address information on outdoor exposure prediction and health effect estimation. In order to achieve the applicability and generalizability of the simulation, we designed our study based on a previous epidemiological study of long-term exposure to particulate matter with a diameter less than or equal to 10 micrometers (PM_10_) and low birth weight (LBW) in Seoul, Korea [28]. We designed our simulation to make our finding applicable to other pollutants than PM_10_ by constructing various exposure scenarios using modified simulation parameters.

## Materials/subjects and methods

Our simulation procedure consists of four steps (Fig. S1): (1) exploratory data analyses to obtain parameters for the underlying distributions of PM_10_ and LBW; (2) generation of true PM_10_ exposure and LBW status; (3) application of incomplete addresses and estimation of mothers’ exposures; and (4) health effect estimation of LBW for PM_10_ and comparison of the performance of health effect estimates by complete and incomplete addresses. We constructed eight environmental scenarios representing different distributions of air pollution of 46,007 mothers, and nine exposure prediction methods under either complete or incomplete residential addresses. The following sub-sections provide detailed information on each step. Further details including formulas are provided in the Supplementary Information.

### Data analysis and parameter acquisition

We obtained parameters to be used for generating exposure and outcome from the exploratory analysis of air quality regulatory monitoring data for PM_10_, geographic variables, and birth certificate data in Seoul, Korea, during 2010. [29, 30] Using the annual average concentrations of PM_10_, we fitted empirical variogram models and estimated mean and variance parameters. Mean parameters were regression coefficients for five geographic variables that were highly associated with particulate matter in Seoul [31]. Three variance parameters include range, partial sill, and nugget that indicate the distance in which spatial correlation exists, spatial variability, and non-spatial variability, respectively. [31–33] For LBW, we obtained birth certificate data from the Statistical Geographic Information Service operated by Statistics Korea and computed the proportion of LBW cases to the total births. [28] To focus on the spatial variation, we restricted our study period to a single year in 2010 and selected 46,007 mothers who had births in 2010.

### Generation of true exposure and outcome

Before generating true exposure and outcome of each mother, we generated the locations of mothers’ homes based on the spatial distribution of the number of births in Seoul (median area and average population in 2010: 21.59 km [2] and 412,520 people), the Capital of South Korea, which is composed of 25 districts, 422 neighbourhoods, and 16,230 census tracts. Because mothers’ addresses in birth certificate data are available at the district level, we treated census tract centroids as mothers’ potential home addresses. We randomly sampled the same number of centroids to those of mothers in each district with the weight of live births across neighbourhoods in each district. These locations were fixed over the simulation.

Assuming that exposure to PM_10_ follows a Gaussian random field with spatial dependency, we generated true annual-average PM_10_ concentrations using mean and variance parameters at all locations (Table 1). These locations included 46,007 mothers’ homes, 37 air quality regulatory monitoring sites, 25 district governmental offices, 422 neighbourhood community centres, 16,230 census tract centroids, and 610 centroids on the 1-km grid in Seoul (Fig. S2). To represent possibly different spatial structures of true PM_10_ annual-average concentrations, we used different combinations of mean and variance parameters and constructed eight environmental scenarios (ES1–ES8). Eight combinations of parameters gave varying contributions of the mean structure, spatial variability, and non-spatial variability of PM_10_ to total variability (Table 1, Fig. S3). While ES1–ES4 was defined based on different spatial correlation structures, ES5-ES8 additionally included different mean structures characterized by five geographic variables that were highly associated with particulate matter in Seoul [31]. ES8 was constructed by the optimal parameters from our data analysis. These various and extended environmental scenarios can also represent different pollutants other than PM_10_.

**Table 1:** Spatial characteristics of eight environmental scenarios (ESs) based on their variability components and variance parameters used for simulating true PM_10_ annual average concentrations.

ES	Variability component^a^			Variance parameter		
	Mean structure	Spatial variability	Non-spatial variability	Nugget	Partial sill	Range (m)
ES1	None	Dominant	Little	1.00	30.94	5885
ES2	None	High	Low	6.86	28.98	9609
ES3	None	Low	High	11.51	34.71	20,355
ES4	None	Little	Dominant	22.00	13.77	27,000
ES5	Moderate	High	Low	1.00	16.98	2524
ES6	Moderate	Low	High	6.86	12.17	4820
ES7	Dominant	High	Low	1.00	10.00	1100
ES8	Dominant	Low	High	6.86	3.60	1004

^a^Spatial characteristics determined by contribution of three variability components (mean structure, and spatial and non-spatial variability) to total variability.

For outcome, we assumed an inverse logit function as the underlying distribution of LBW. Then, we generated LBW status of mothers using simulated true PM_10_ concentrations, the proportion of LBW cases, and the effect estimate of LBW for PM_10_ obtained from our previous study [28].

### Exposure prediction

Using simulated PM_10_ at 37 regulatory monitoring sites, we applied nine prediction methods to estimate mothers’ individual exposure to PM_10_ by complete and incomplete address conditions (Table S1). When mothers’ complete home addresses were available, we applied four prediction methods commonly used in previous studies [6, 10, 11]. In the nearest monitor (NM) and inverse distance weighted average (IDWA) methods solely based on (“simulated”) measurements, we assigned PM_10_ at the monitoring site nearest to a home of each mother and averaged across the sites weighted by inverse squared Euclidean distance from each home, respectively. The other two approaches employed modelling approaches including geographic characteristics that represent direct or indirect pollution sources. Land use regression (LUR) includes these characteristics as predictors in regression equations. Universal kriging (UK) additionally includes spatial correlation as a geostatistical method that optimally derives interpolated concentrations based on mean structure and spatial correlation. We built LUR and UK models using the same five geographic variables to those used in the generation of true PM_10_ (See the “Data Analysis and Parameter Acquisition” section). Out of the 37 regulatory monitoring sites in Seoul, we used underlying PM_10_ concentrations from 25 urban-background sites for NM, IDWA, and area averaging (AA), and from all 37 sites including 12 urban roadside sites for LUR and UK. We predicted PM_10_ concentrations of mothers using estimated regression and/or variance parameters in LUR and UK along with geographic variables at mothers’ homes.

When address data were assumed to be incomplete and available at the district level, we applied one measurement-based and four model-based prediction approaches. In AA as a measurement-based approach, we computed the average concentration across all monitoring sites in a district to all mothers living in the same area as often used in earlier cohort studies of air pollution [34, 35]. Since the regulatory monitoring network in Seoul had one urban-background site in every district, we treated the PM_10_ concentration at a single site as a special case of AA. In addition, we applied UK to compute area-level representative exposure and developed four approaches. Here, we assumed when a pointwise prediction model is available but complete address data are unavailable, a preferred option could be the aggregation of predictions at many representative points [15, 36]. We used three representative locations for aggregation: 422 neighbourhood community centres (UKNA), 16,230 census tract centroids (UKCA), and 610 1-km grid coordinates (UKGA). We predicted PM_10_ concentrations using UK at these three types of locations, computed district averages, and assigned to the mothers living in the same districts. UKNA and UKCA predictions represent population exposure at the fine spatial scale, whereas UKGA predictions focus on spatially-representative exposure based on spatially even distribution of PM_10_. We also used predictions at 25 district governmental offices without aggregation (UKD) for comparison.

### Health effect estimation and comparison of properties

Using true and predicted PM_10_ as well as true LBW status of mothers, we estimated the health effects of LBW for PM_10_ using logistic regression. Then, we repeated the whole procedure from exposure generation to health effect estimation 1000 times, and computed properties of health effect estimates over 1,000 simulations as bias, root mean square error (RMSE), average standard error (ASE), coverage probability (CP), and true positive rate (TPR). CP was computed as the proportion of the simulations where the 95% confidence intervals of health effect estimates contain the true effect. TPR was the ratio of the number of simulations that provide significantly positive health effect estimates for each predicted PM_10_ (*p* value < 0.05) to those for true PM_10_. While bias, RMSE, ASE, and CP aim to evaluate the accuracy or uncertainty of the estimates, TPR can provide the insight into statistical power. Finally, we compared the health effect estimate properties between complete and incomplete addresses depending on the exposure prediction method and pollution environment.

## Results

### True and predicted PM_10_

**Fig. 1:**
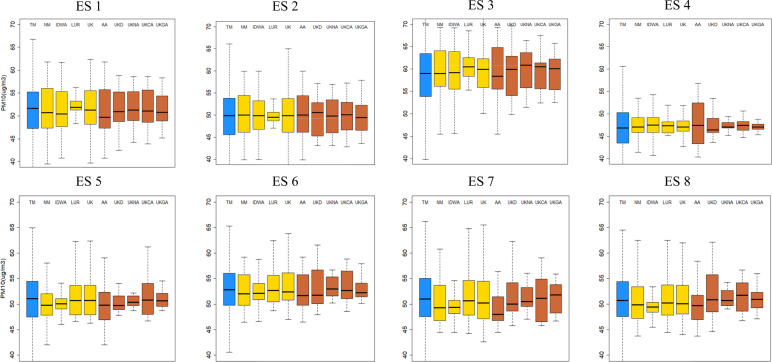
Box-plots of true (TE true exposure) and predicted (NM nearest monitor, IDWA inverse distance weight average, LUR land-use regression, AA area average, UK universal kriging, UKD UK prediction at governmental offices, UKNA district average based on UK predictions at 422 neighborhood community centers, UKCA district average of UK predictions at 16,230 census tract centroids, UKGA district average of UK predictions at 610 1-km grid coordinates) annual-average PM_10_ concentrations at home addresses of 46,007 mothers by eight environmental scenarios (ES1–ES8) in the 1st simulation (blue boxes for true exposure; yellow and red boxes for predicted exposure with complete and incomplete addresses, respectively).

Table S2 and Fig. 1 summarize true and predicted annual-average PM_10_ concentrations at home addresses of 46,007 mothers by different ESs and exposure prediction methods. Mothers’ PM_10_ concentrations predicted at their homes (mean = 47.25–60.52 µg/m^3^, standard deviation (SD) = 1.04–6.45 µg/m^3^) were generally similar on average but less variable compared to true concentrations (46.90–58.55 µg/m^3^, 4.66–6.45 µg/m^3^). Variability was even smaller when address information was restricted to the district (SD = 1.04–4.56 µg/m^3^), compared to complete addresses (1.56–6.45 µg/m^3^). This pattern was similar across eight ESs with the smallest mean and variability in ES4 where there is no spatial variability in exposure. Across nine prediction methods, the correlation with true exposure was generally higher in UK and UKCA (Pearson correlation coefficient = 0.26–0.70), with complete and incomplete address data respectively, compared to the other prediction methods (0.00–0.65) across all ESs except ES4 that showed little correlation (Table S2, Fig. S3). NM, IDWA, AA, and UKD gave relatively high correlation and slopes close to 1 when there was no mean structure with some spatial correlation in ES1-ES3, but low correlation otherwise. In contrast, LUR provided high correlations (0.60–0.65) when there was a dominant mean structure as shown in ES7 and ES8, but low correlations less than 0.1 without a mean structure.

### Health effect estimate properties by address availability

Performance of effect estimates of LBW for true and predicted annual-average PM_10_ concentrations became worse when data availability for PM_10_ or address was limited. Table 2 shows the average relative risk, as well as bias, RMSE, ASW, and CP of health effect estimates in four ESs including ES2, ES3, ES5, and ES8 where different exposure environments are more distinct: Tables S3 and S4, and Figs. S4 and S5 show all eight scenarios. Bias and RMSE tended to increase using predicted exposure compared to true exposure, while there was a slightly larger increase with incomplete addresses than with complete addresses. Larger bias is also seen in scatter plots of health effect estimates of predicted exposures against those of true exposures in Fig. S6. Performance varied more across different prediction methods and environmental scenarios under complete addresses than incomplete addresses. Regardless of address availability, CPs were close to 0.95 (Fig. S7). TPR was generally lower with incomplete addresses than complete addresses (Fig. S8).

**Table 2:** Estimated relative risks and their properties (Bias, RMSE, ASE, and CP) of health effect estimates of true and predicted PM_10_ concentrations on low birth weight over 1,000 simulations by address availability, exposure prediction methods, and environmental scenarios (ES2, ES3, ES5, and ES8).

		ES2					ES3				
Address availability	Exposure prediction	$\widehat {RR}$^a^	Bias^b^	RMSE^c^	ASE^d^	CP^e^	$\widehat {RR}$	Bias	RMSE	ASE	CP
	TE	1.0029	−0.04	1.48	0.78	0.95	1.0036	0.03	1.48	0.75	0.95
Complete	UK	1.0030	−0.03	2.41	1.45	0.94	1.0028	−0.05	2.72	1.58	0.94
Incomplete	AA	1.0018	−0.15	1.55	0.83	0.94	1.0019	−0.14	1.56	0.80	0.95
	UKD	1.0025	−0.08	2.42	1.41	0.94	1.0024	−0.09	2.75	1.62	0.94
	UKNA	1.0032	−0.01	3.27	1.95	0.94	1.0040	0.07	4.08	2.67	0.94
	UKCA	1.0033	0.00	2.84	1.74	0.94	1.0038	0.05	3.32	2.00	0.95
	UKGA	1.0042	0.09	3.83	2.38	0.94	1.0037	0.04	4.83	3.63	0.94
		**ES5**					**ES8**				
**Address availability**	**Exposure prediction**	$< Emphasis Type="BoldItalic" > \widehat {RR} < /Emphasis >$	** Bias**	**RMSE**	**ASE**	**CP**	$< Emphasis Type="BoldItalic" > \widehat {RR} < /Emphasis >$	**Bias**	**RMSE**	**ASE**	**CP**
	TE	1.0036	0.03	1.50	0.78	0.95	1.0033	0.00	1.40	0.71	0.95
Complete	UK	1.0034	0.01	2.06	1.10	0.95	1.0032	−0.01	1.73	0.88	0.96
Incomplete	AA	1.0014	−0.19	1.76	0.95	0.95	1.0008	−0.25	2.04	1.05	0.95
	UKD	1.0017	−0.16	2.02	1.07	0.94	1.0009	−0.24	1.93	1.03	0.95
	UKNA	1.0037	0.04	3.10	1.68	0.94	1.0039	0.06	4.07	2.14	0.94
	UKCA	1.0038	0.01	2.33	1.26	0.94	1.0035	0.02	2.23	1.16	0.96
	UKGA	1.0037	0.05	3.71	2.04	0.95	1.0022	−0.11	3.31	1.73	0.95

^a^Estimated Average Relative Risk (True Relative Risk = 1.0033).

^b^Bias multiplied by 100.

^c^Root mean square error (RMSE) multiplied by 100.

^d^Average standard error (ASE) multiplied by 100.

^e^Coverage probability (CP) of 95% confidence interval.

^f^TE: true exposure; UK: universal kriging_;_ AA: area-average UKD: UK prediction at governmental offices without aggregation; UKNA: district average based on UK predictions at 422 neighbourhood community centres; UKCA: district average of UK predictions at 16,230 census tract centroids; UKGA: district average of UK predictions at 610 1-km grid coordinates.

### Health effect estimate properties by exposure prediction

All prediction methods under complete addresses, and AA and UKD under incomplete addresses mostly showed negative bias indicating under-estimated health effects (Table 2, S3, and S4). Bias was particularly large for NM, AA, and UKD that relied on the measurement or prediction at a single monitoring site to assess individual exposure. However, UKD using predictions estimated at governmental offices that were largely located in highly populated areas gave smaller bias than NM and AA based on measurements at regulatory monitoring sites. When address data were fully available, UK gave lower bias and RMSE than other approaches across all ESs. This good performance was notably prominent in ES5 to ES8 which includes a mean structure. NM tended to provide small RMSE and ASE, but relatively large negative bias, while IDWA gave relatively small bias but large RMSE and ASE. LUR provided good performance only when there is a mean structure in the true environment (ES5 to ES8). When address data were limited to the district, three UK-based district averages showed much smaller bias with either direction and slightly higher RMSE and ASE compared to the other two prediction methods of AA and UKD. Among these three approaches, UKCA as the exposure averaged over a large number of population-representative points at the fine spatial scale showed better performance than UKNA and UKGA based on coarse spatial-scale population-representative points and spatially-representative points, respectively. TPR was also generally higher in UK and UK-based district averages (Fig. S8).

### Health effect estimate properties by environmental scenarios

Better performance of UK and UKCA under complete and incomplete address conditions, respectively, were consistent across all eight ESs (Tables S3–S5, and Figs. S5–S8). Large bias in NM, AA, and UKD, and large uncertainty in UKNA and UKGA, possibly resulting from small variability in PM_10_, were also consistent across all ESs. ES4 showed large bias and RMSE and small TPR across all prediction methods.

## Discussion

This study focused on the impact of limited availability of address data on health effect estimation compared to complete availability. After hypothesizing that address data availability affects health effect analysis of predicted exposure, we explored the impact of address availability on the performance of health effect estimates depending on exposure prediction methods and environmental scenarios based on the real-world example of the association between PM_10_ and LBW. Eight environmental scenarios represented various pollution environments related to the different contributions of geographic features and spatial dependency. Furthermore, nine prediction methods exhibited commonly applied approaches of individual exposure assessment given limited monitoring data with and without additional limitation in address data. Our findings showed that when address data are limited, individual exposure modelled by geographic characteristics and averaged across population-representative points, as shown in UK-based averaging, can provide comparable accuracy in health effect estimation to those using complete address information. This improved accuracy was prominent compared to other exposure prediction approaches and generally consistent across different environmental scenarios.

Our simulation study intended to answer an important question that can help inference of epidemiological studies of air pollution relying on limited address data of subjects. Even though many recent studies developed advanced exposure prediction models and allowed the estimation of air pollution concentrations at people’s homes or workplaces, the benefit of this advance could be limited in many epidemiological studies that are based on existing cohorts and/or administrative health data with incomplete address information. As recent epidemiological studies of air pollution expanded their spatial and temporal coverage to the national or regional scale and to the past several decades, the reliance on existing health data lacking complete address information has become even greater. However, there have been few studies that investigated the impact of limited address information on health analysis and provided realistic guidance. For example, recent two nationwide cohort studies including limited address data applied prediction models to estimate individual-level long-term PM_2.5_ concentrations at zip code-level addresses of Medicare [4] and Canadian Census Health and Environment [37] cohort participants, and reported the association with total mortality. Our simulation findings of negative bias using single points in administrative areas suggest the possibility of underestimated health effects in such studies.

Our findings generally showed that kriging-based approaches gave good performance in health effect estimates consistently across different air pollution environments, when individual air pollution measurements are not available. While UK showed better performance compared to other prediction approaches when complete address data are available, UK averaging approaches outperformed with individual address data limited to the district. A possible explanation is that UK modelled by using both mean and variance structures well characterizes air pollution conditions at people’s residences even when there is no mean structure [38]. In addition, employment of population-representative locations and the following averaging process under the unavailability of precise residential addresses possibly minimized the impact of exposure misclassification. Bias was the smallest and also non-systematic as opposed to other prediction methods that consistently gave negative bias. Out of three UK averaging approaches, UKCA based on UK predictions at census tract centroids gave the lowest RMSE and ASE which were comparable to those of other prediction approaches under the complete address condition. UKCA also showed comparable TPR to those with complete addresses, while it was less likely to detect statistically significant health effect estimates overall with incomplete addresses. However, the benefit of UK-based averaging could be reduced, when we use predictions at the locations including those poorly represented for population as shown in UKGA. CPs close to 95% nominal level across all prediction models might have induced statistically non-significant health effect estimates. However, our finding of the true positive rate distinctively higher in kriging-based approaches compared to others indicates the advantage of kriging.

All prediction methods except for UK-based averaging generally showed underestimated health effects given limited PM_10_ or address data. This underestimation can be explained by exposure measurement error derived by poor characterization of individual exposure in prediction models [39, 40]. In our simulation, prediction methods heavily relying on a mean structure such as LUR gave greater underestimation when there is no mean structure in true exposure scenarios, while simple prediction approaches using measurements only shown as NM and IDWA gave larger underestimation when there is a mean structure. Prediction methods using a single location based on the nearest monitor (NM), or district governmental office (UKD) also gave larger negative bias than other methods. Bias was larger in AA and NM based on regulatory monitoring sites than UKD using population-representative locations. In addition, poor assessment of individual exposure can result from poor representativeness of prediction points used for averaging. Our study showed increased positive or negative bias in UKGA using grid coordinates than UKCA based on census tract centroids. Previous simulation studies reported that measurement error derived by a spatial misalignment between regulatory monitoring sites and people’s residences affected misspecification of prediction models and resulted in positive or negative bias in following health effect analysis [41–43]. Our findings of large bias in AA, NM, LUR, and UKGA possibly suggest the impact of this classical-type measurement error resulted from poor model specification. Relatively large uncertainty in kriging suggests the impact of the Berkson-type measurement error driven by spatial smoothing [42].

Our simulation using various environmental scenarios and parameters obtained from data analyses suggests a possible generalization of our findings to other pollutants and/or study areas. Although we focused on PM_10_ which is well known as a regional pollutant with relatively weak mean structure and large spatial correlation, we constructed seven environmental scenarios by assuming different spatial structures in addition to the ES8 based on the parameters estimated directly from the regulatory monitoring data in Seoul. This variation of spatial structure possibly represents more local or regional pollutants with different impacts of local sources or spatial homogeneity, and allows us to apply our findings to other pollutants such as PM_2.5_ and NO_2_ and/or different regions. In addition, our reliance on real-world data can improve the practical applicability of our simulation findings.

Our study includes several limitations to be further investigated in future research. First, we focused on ambient exposure and did not consider the impact of indoor exposure. However, this impact could be small for PM which showed relatively high infiltration compared to other pollutants [44]. Besides, our application of diverse environmental scenarios including locally heterogeneous exposure may also represent indoor and/or personal exposure. Second, we created mothers’ residential addresses using census tract centroids and assumed them fixed over the simulation. Future studies that apply real addresses of participants and/or incorporate mobility should investigate the sensitivity of our findings. Third, we did not consider multi-pollutant models and correlated exposure measurement error could affect bias [45]. Future studies should investigate this impact in cohort-study design. Lastly, we used low birth weight and logistic regression. Future studies should confirm whether our suggestions are consistent with different health outcomes and health analysis models.

In conclusion, this simulation study suggests that exposure prediction approaches well representing geographic environments and supplemented with population-representative prediction locations can improve accuracy in health effect estimation when complete individual address data are not available. Our findings also provide guidance for a preferred approach to improve the inference in future large-scale epidemiological studies of long-term air pollution.

## Supplementary information

Supplementary information

## Data Availability

Simulated data and sample code are available from the github page of the first author YB Jun (http://github.com/junpeea) on reasonable request. Also, supplementary results are provided in the supplemental material.
