# Correction to: Impact of limited residential address on health effect analysis of predicted air pollution in a simulation study

**DOI:** 10.1038/s41370-022-00432-x

**Published:** 2022-03-31

**Authors:** Yoon-Bae Jun, Insang Song, Ok-Jin Kim, Sun-Young Kim

**Affiliations:** 1 Department of Statistics, Iowa State University, 1212, Snedecor Hall, 2438 Osborn Dr, Ames, IA 50011 USA; 2 Department of Geography, University of Oregon, Eugene, OR 97403 USA; 3 Environmental Health Research Department, Environmental Health Research Division, National Institute of Environmental Research, Incheon, Korea; 4 Department of Cancer Control and Population Health, Graduate School of Cancer Science and Policy, National Cancer Center, Goyang-si, Gyeonggi-do Korea

Correction to: *Journal of Exposure Science and Environmental Epidemiology* 10.1038/s41370-022-00412-1, published online 26 January 2022

The article “Impact of limited residential address on health effect analysis of predicted air pollution in a simulation study”, written by Yoon-Bae Jun, Insang Song, Ok-Jin Kim, Sun-Young Kim, was originally published electronically on the publisher’s internet portal on 26 January 2022 without open access. With the authors’ decision to opt for Open Choice the copyright of the article changed on 09 February 2022 to © The Author(s) 2022 and the article is forthwith distributed under a Creative Commons Attribution 4.0 International License, which permits use, sharing, adaptation, distribution and reproduction in any medium or format, as long as you give appropriate credit to the original author(s) and the source, provide a link to the Creative Commons licence, and indicate if changes were made. The images or other third party material in this article are included in the article’s Creative Commons licence, unless indicated otherwise in a credit line to the material. If material is not included in the article’s Creative Commons licence and your intended use is not permitted by statutory regulation or exceeds the permitted use, you will need to obtain permission directly from the copyright holder. To view a copy of this licence, visit http://creativecommons.org/licenses/by/4.0/.

