# Supplementary material for: Impact of limited residential address on health effect analysis of predicted air pollution in a simulation study
Source: J Expo Sci Environ Epidemiol. 2022 Jan 26;32(4):637–43. doi: 10.1038/s41370-022-00412-1 (PMC9349037; doi:10.1038/s41370-022-00412-1)
Supplement: Supplementary file 1 — Supplementary information [file 41370_2022_412_MOESM1_ESM.docx]

**Supplementary Information**

**Impact of limited address data on health effect analysis of long-term air pollution in a simulation study**

Yoon-Bae Jun, Insang Song, Ok-Jin Kim, Sun-Young Kim

**Details of simulation procedure**……………………………………………………………2

**List of Figures**

**Figure S1.** Summary flow charts of our simulation study……………………..……………..8

**Figure S2.** Maps of study area ………………........................................................................9

**Figure S3.** Maps of true simulated annual-average PM_10_ concentrations…..........................10

**Figure S4.** Scatter plots of true and predicted annual-average PM_10_ concentrations………11

**Figure S5.** Bar plots of bias of health effect estimates……………………………………...12

**Figure S6.** Scatter plots of health effect estimates ………………………………………….14

**Figure S7.** Bar plots of root mean square error, average standard error, and coverage probability of health effect estimates…………………………………………..………..…...16

**Figure S8.** Bar plots of true-positive rate of health effect estimates…………………...……17

**List of Tables**

**Table S1.** Table of different exposure prediction methods and their illustrations ………….18

**Table S2.** Mean and standard deviation of true and predicted PM_10_………………………...20

**Table S3.** Correlation and regression coefficients of true and predicted PM_10_ ………….....22

**Table S4.** Properties of health effect estimates of true and predicted PM_10_ (ES1-ES4) …...24

**Table S5.** Properties of health effect estimates of true and predicted PM_10_ (ES5-ES8)........26

**Details of simulation procedure**

Data Analysis and Parameter Acquisition

We obtained parameters to be applied to our simulation from the exploratory analysis of air quality regulatory monitoring data for PM_10_, geographic variables, and birth certificate data in Seoul, South Korea, during 2010. Hourly PM_10_ measurement data in the regulatory monitoring network were obtained from the National Institute of Environmental Research. The air quality regulatory monitoring network in Seoul includes 25 urban-background and 12 urban-roadside sites in 2010. Urban-background sites are located in heavily populated residential areas with the aim of assessing the population level of exposure; one monitoring site is deployed in each of the 25 districts in Seoul^1^. In contrast, urban-roadside sites are located next to large and busy major roads for monitoring air pollution affected by traffic emissions. Using hourly measurements, we computed daily averages at each site for the days when at least 75% of hourly measurements are available. Then, the annual average concentrations were computed at each site where there is at least one daily measurement in each of the 10 months and in any 45 consecutive days^2^.

Using annual average concentrations of PM_10_, we fitted variogram models and estimated mean and variance parameters. Three variance parameters include range, partial sill, and nugget that indicate the distance in which spatial correlation exists, spatial variability, and non-spatial variability, respectively^3^. Five mean parameters were regression coefficients of five geographic variables that were most related to fine particulate matter less than or equal to 2.5 micrometer per diameter (PM_2.5_) in our previous study for Seoul^4^. The data sources of geographic variables and computation procedure in Geographic Information System were described elsewhere^5^. These variables include the length of major roads in a 100- m circular buffer, the proportion of water surface land use in 500 m, the number of construction companies in 1,000 m, the distance to the nearest bus stop, and the number of employees in construction industries in 100 m.

We obtained birth certificate data from the Statistics Korea (http://kosis.kr/eng/). Term LBW babies were defined as singleton live births between 37 to 41 weeks with less than 2.5 kilograms^6^. We computed the proportion of LBW cases to the total births (0.016) and used in the simulation as the true LBW proportion. The underlying effect estimate of PM_10_ on LBW (0.003) was obtained from our previous study of the association between PM_10_ and LBW in 549,270 non-employed mothers residing in Seoul, for 2002-2012^6^. To focus on the spatial variation, we restricted our study period to a single year in 2010 and selected 46,007 mothers who had births in 2010.

Generation of True Exposure and Outcome

Because mothers’ residential addresses in birth certificate data are available at the district level, we generated the locations of mothers’ homes based on the spatial distribution of the number of births. Seoul, the Capital of South Korea, is composed of 25 districts (median area and average population in 2010: 21.59 km^2^ and 412,520) and 422 neighbourhoods (8.69 km^2^ and 24,323). There are 16,230 census tracts (0.02 km^2^ and 821) as the smallest census territorial unit nested within neighbourhoods. We treated census tract centroids as mothers’ potential home addresses and randomly sampled the centroids for the mothers in each district with the weight of the numbers of live births across neighbourhoods of each district. Census tract centroids are considered socio-demographically homogeneous within a census tract area and different across areas. We treated these locations fixed over the simulation.

We generated true annual average PM_10_ concentrations, as true exposures to PM_10_, at all locations including 46,007 mothers’ homes, 37 air quality regulatory monitoring sites, and the centroids of 25 district governmental offices, 422 neighbourhood community centers, 16,230 census tract centroids, and 610 centroids on the 1-km grid in Seoul. As shown in the equation 1, we assumed that the true exposure to PM_10_ at a location s, $X(s)$, follows Gaussian random field, and is partitioned into three components of global mean,$\mu$, spatial error,$\eta$, and non-spatial error,$\varepsilon$.

|  | $X\left( s \right)= \mu\left( \boldsymbol{Z}\left( s \right);\boldsymbol{\gamma} \right)+ \eta\left( s;\sigma^{2},\phi\right)+ \varepsilon(s;\tau)$ | (1) |
| --- | --- | --- |

$\mu$ is a linear function of predictors, $\boldsymbol{Z}\left( s \right)$, characterized by mean parameters, $\boldsymbol{\gamma}$. These predictors include five geographic variables that were most associated with particulate matter concentrations in our previous study^4^. $\eta$ is a spatial random process characterized by partial sill, $\sigma^{2}$, and range, $\phi$, that represent spatial variability and the distance in which spatial correlation exists, respectively. $\varepsilon$ is a non-spatial random process indicating measurement error and characterized by nugget, $\tau^{2}$, representing non-spatial variability. Consequently, the total variability of the true PM_10_ annual-average concentrations,$Var(X)$, can be partitioned into global trend variability, $Var(\mu)$, partial sill, $\mathrm{Var}\left( \eta\right)= \sigma^{2}$, and nugget, $\mathrm{Var}\left( \varepsilon\right)=\tau^{2}$.

To represent possibly different spatial structures of true PM_10_ annual average concentrations, we constructed eight environmental scenarios (ES1-ES8) based on varying contributions of the three components of PM_10_ to total variability (Table 1, Figure S1). ES1 to ES4 has a constant mean (i.e. $\mathrm{Var}\left( \mu\right)=0$) and different variance parameters of range, partial sill, and nugget which represent different contribution to spatial and non-spatial variability. From ES1 to ES4, the contribution of spatial variability decreases while the contribution of non-spatial variability increases. The other four scenarios (ES5-ES8) include mean structures characterized by five geographic variables (i.e. $\mathrm{Var}\left( \mu\right)\neq0$) in addition to different combination of variance parameters. Whereas ES5 and ES6 have intermediate contribution of mean structure, ES7 and ES8 have dominant contribution. ES5 and ES7 contain more spatial variability than non-spatial variability, while ES6 and ES8 comprise more non-spatial variability than spatial variability. ES8 was constructed by using the parameters that best represent the data, indicating the most similar environment to Seoul.

LBW status of mothers were generated based on the proportion of LBW cases obtained by our exploratory data analysis (see the Data Analysis and Parameter Acquisition section), simulated true PM_10_ concentrations (see the Exposure Generation section), and the effect estimate of LBW for PM_10_ obtained by our previous study^6^. We assumed that LBW probability of a mother, p, follows an inverse logit function of true baseline LBW rate ($\beta_{0}$) and the true effect estimate of LBW ($\beta_{1}$) for individual PM_10_ concentration (X), as shown in the equation 2.

|  | $p=exp\left( \beta_{0}+\beta_{1}X \right) / (1+exp\left( \beta_{0}+\beta_{1}X \right))$. | (2) |
| --- | --- | --- |

Then, given LBW probability of each mother, p, we randomly generated LBW status from Bernoulli distribution.

Health Effect Estimation and Comparison of Properties

Using true or predicted PM_10_ and true LBW status of mothers, we estimated health effects of LBW, and computed properties of health effect estimates ($\grave{\beta}_{sim}$) over 1,000 simulations to evaluate accuracy of effect estimates depending on different scenarios for address availability, exposure prediction methods, and pollution environments. These properties include bias ($\frac{1}{1000}\sum_{sim=1}^{1000} (\grave{\beta}_{sim}-\beta)$), root mean square error (RMSE) ($\sqrt{\frac{1}{1000}\sum_{sim=1}^{1000} {(\grave{\beta}_{sim}-\beta)}^{2}}$), average of standard error (ASE) ($\frac{1}{1000}\sum_{sim=1}^{1000} \sqrt{Var(\grave{\beta}_{sim})}$), and coverage probability (CP) ($\frac{1}{1000}\sum_{sim=1}^{1000} Ind[\grave{\beta}_{sim}-1.96\times\sqrt{\mathrm{Var}(\grave{\beta}_{sim})}\leq\beta\leq\grave{\beta}_{sim}+1.96\times\sqrt{\mathrm{Var}\left( \grave{\beta}_{sim} \right)}$], where $Ind[condition]=1$ if the condition is true, and $Ind[condition]=0$ otherwise). RMSE and ASE indicate the uncertainty of effect estimates on average, while CP presents the probability of including the true effect estimate within estimated 95% confidence intervals. In addition, to explore the impact of address availability on statistical power to detect the true association, we computed the true positive rate (TPR), which is the ratio of the number of simulations that provide significantly positive effect estimates for each predicted PM_10_ (p-value < 0.05) to the number of significant simulations for true PM_10_.

References

1. National Institute of Environmental Research. 2016 NIER Annual Report. (2017).

2. Yi, S.-J. *et al.* Association between Exposure to Traffic-Related Air Pollution and Prevalence of Allergic Diseases in Children, Seoul, Korea. *BioMed Research International* (2017).

3. Cressie, N. *Statistics for Spatial Data*. (Wiley-Interscience, 2015).

4. Min, K. D., Kwon, H. J., Kim, K. S. & Kim, S. Y. Air pollution monitoring design for epidemiological application in a densely populated city. *Int. J. Environ. Res. Public Health* **14**, 1–12 (2017).

5. Eum, Y., Song, I., Kim, H.-C., Leem, J.-H. & Kim, S.-Y. Computation of geographic variables for air pollution prediction models in South Korea. *Environ. Health Toxicol.* **30**, (2015).

6. Choe, S.-A., Jang, J., Kim, M. J., Jun, Y.-B. & Kim, S.-Y. Association between ambient particulate matter concentration and fetal growth restriction stratified by maternal employment. *BMC Pregnancy Childbirth* **19**, 246 (2019).

Figure S1 Summary flow charts of four steps in our simulation study

Figure S2. Maps of (a) study area, Seoul, the Capital of South Korea, (b) 25 urban-background regular monitoring sites (red dots) in 25 districts, Seoul, and (c) neighborhood community centers (light blue) and census tracts centroids (light purple) in a red-lined district in (b), and (d) 1-km grid coordinates (green)


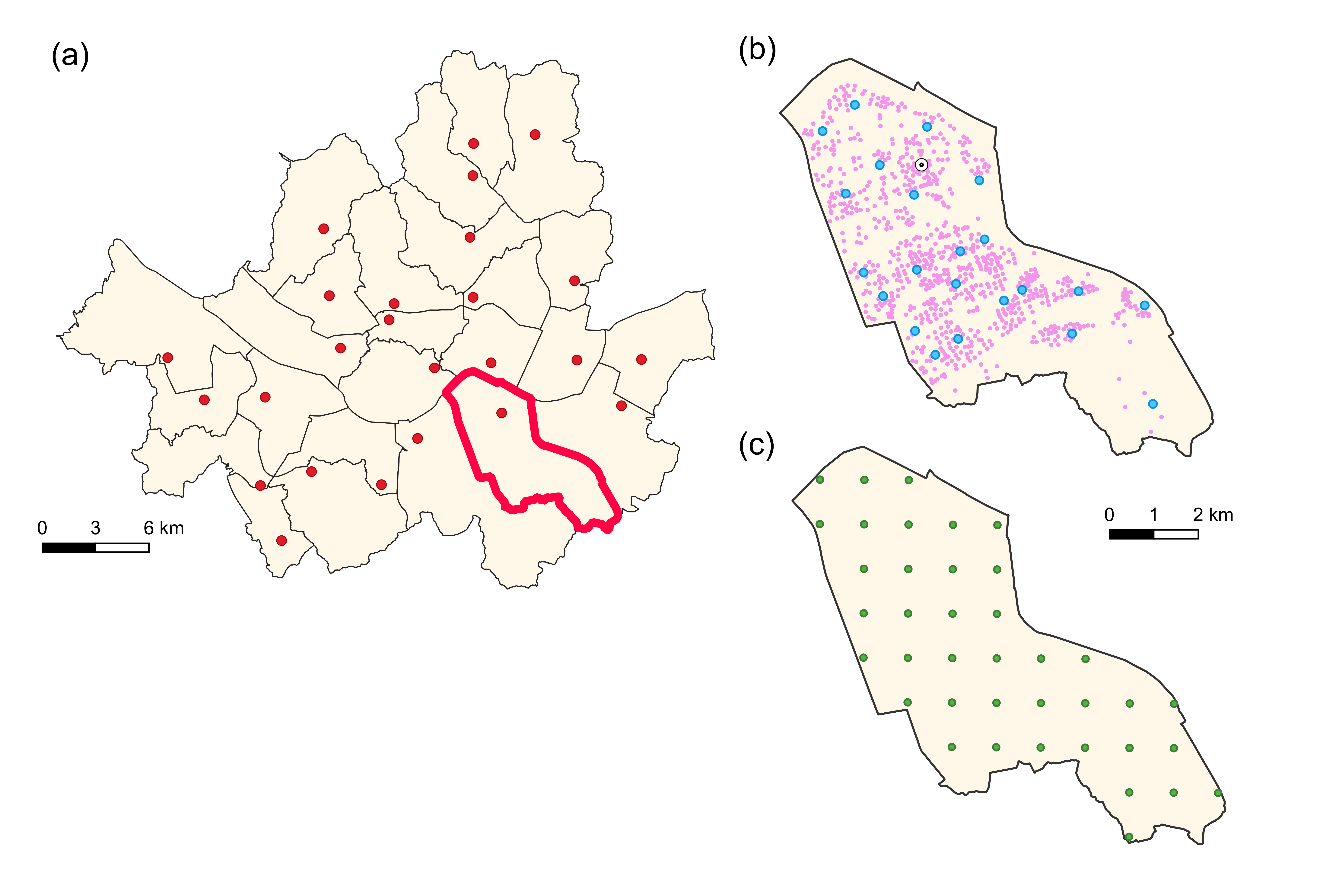


(b)

(c)

(d)


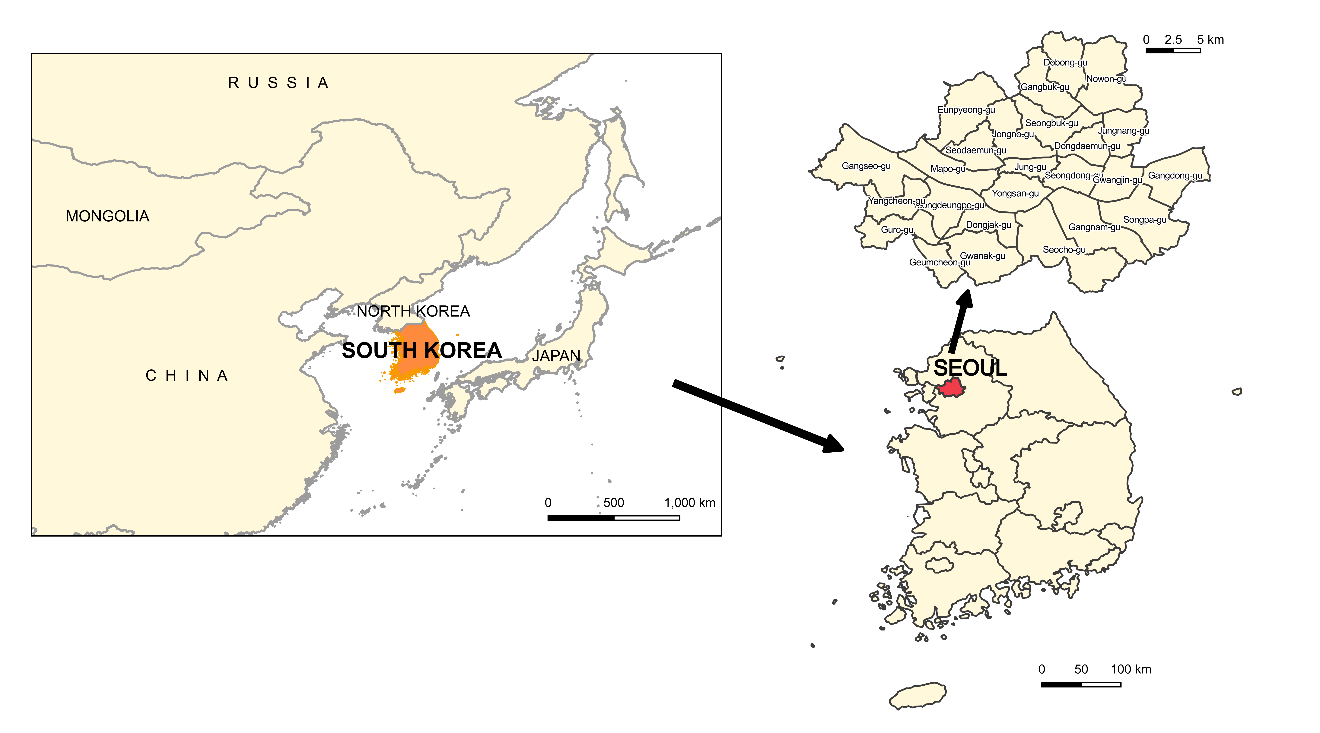


(a)

**Figure S3.** Maps of true simulated annual-average PM_10_ concentrations by eight environmental scenarios (ESs) in Seoul, Korea

| **ES1** | **ES2** | **ES3** | **ES4** |
| --- | --- | --- | --- |
| 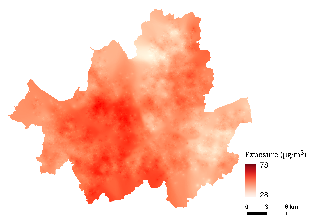 | 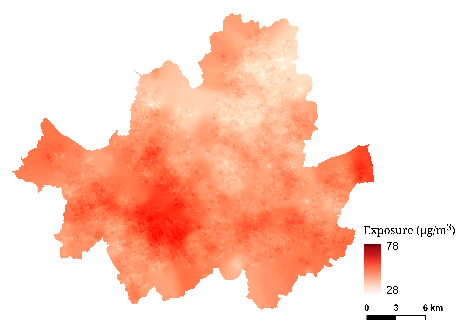 | 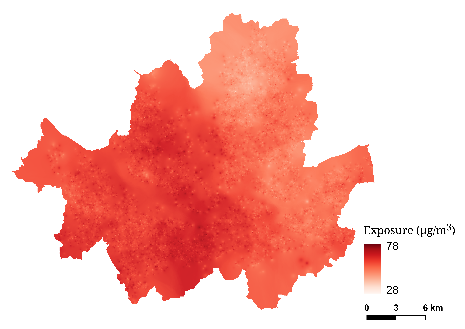 | 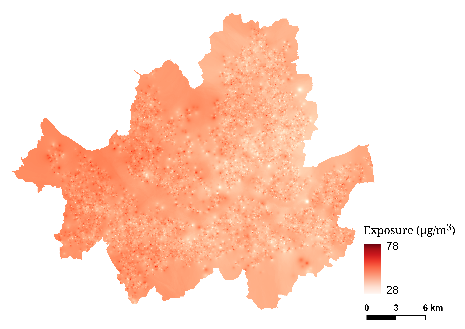 |
| **ES5** | **ES6** | **ES7** | **ES8** |
| 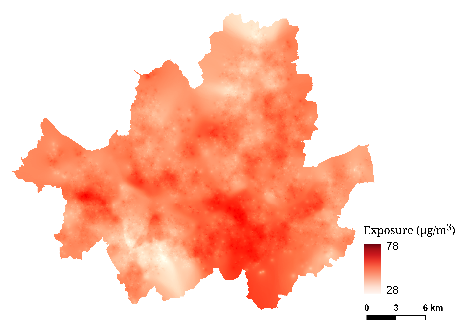 | 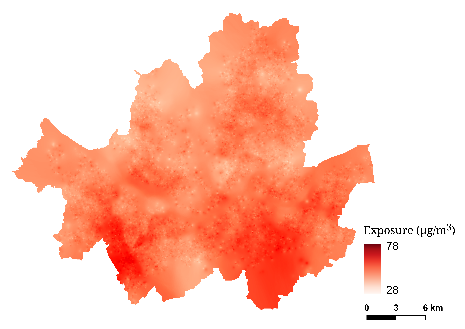 | 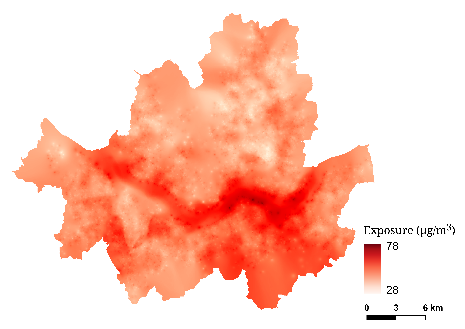 | 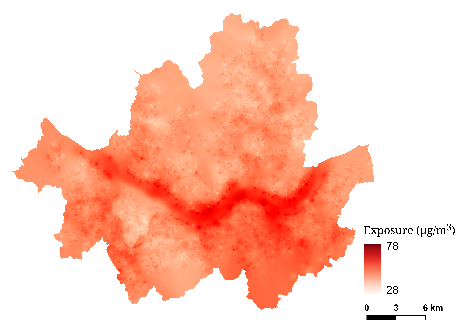 |

**Figure S4.** Scatter plots of true (x-axis) and predicted (y-axis) annual-average PM_10_ concentrations at home addresses of 46,007 mothers in the 1st simulation (blue and red lines for best-fitted and 45 degree lines, respectively)

|  | ES1 | | ES2 | | ES3 | | ES4 | | ES5 | | ES6 | | ES7 | | ES8 | |
| --- | --- | --- | --- | --- | --- | --- | --- | --- | --- | --- | --- | --- | --- | --- | --- | --- |
| NM | | 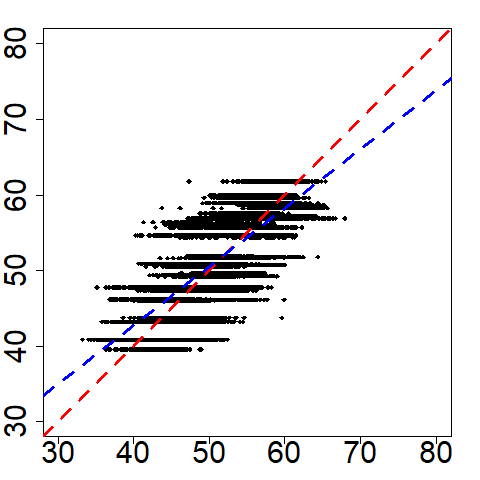 | | 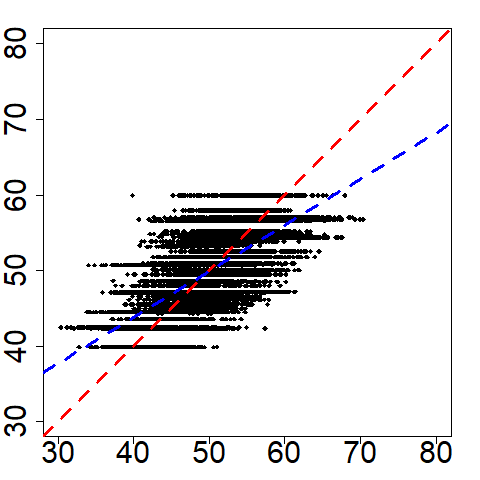 | | 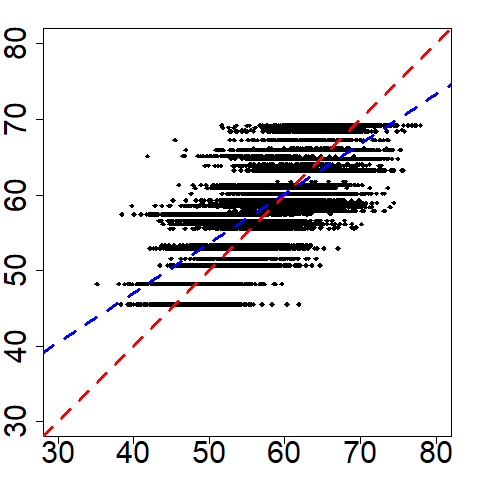 | | 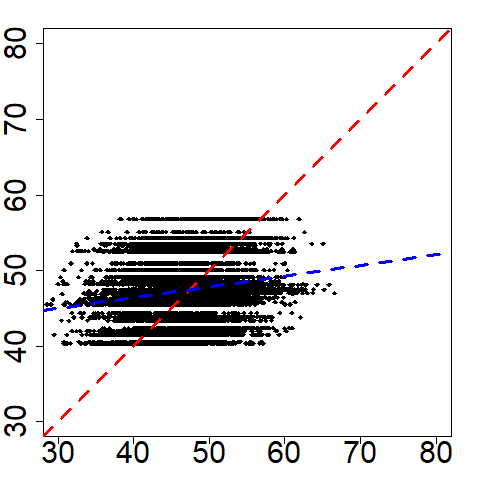 | | 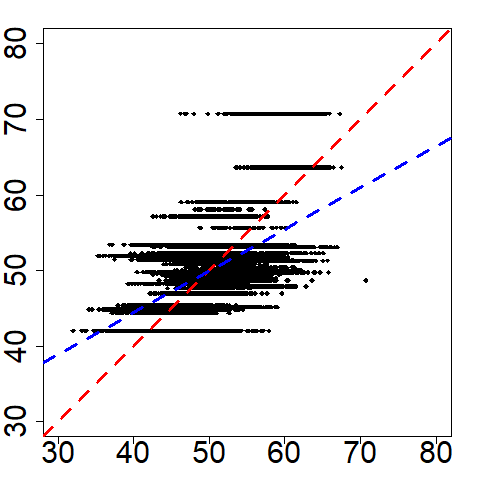 | | 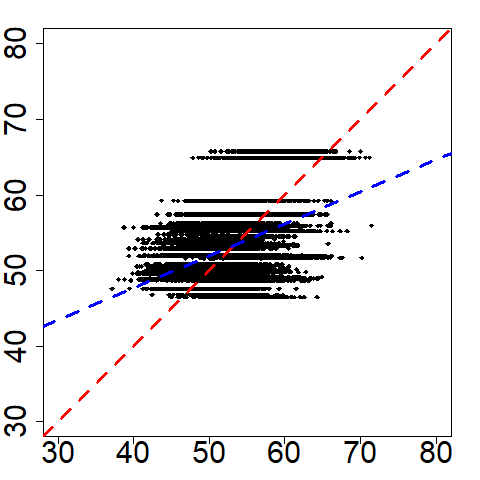 | | 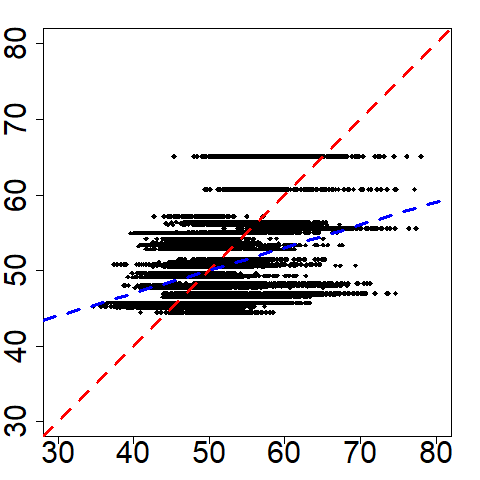 | | 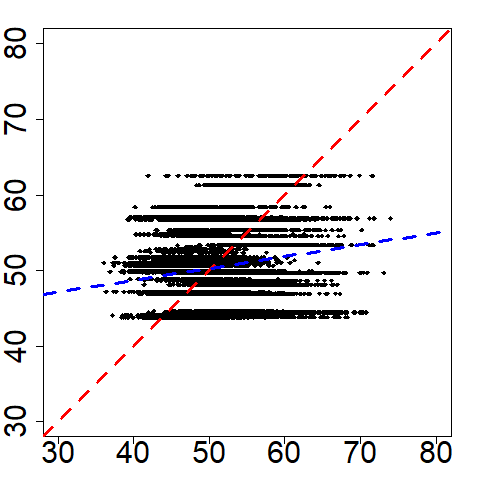 |
| IDWA | | 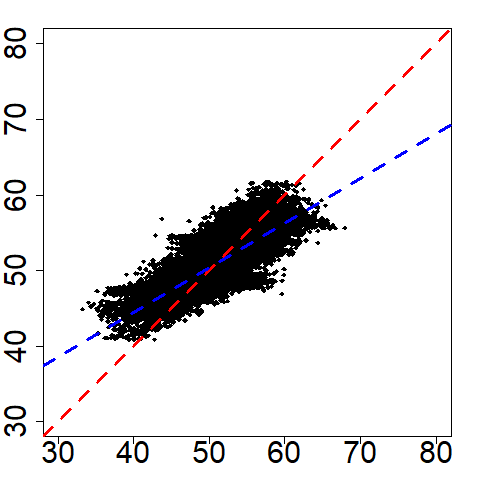 | | 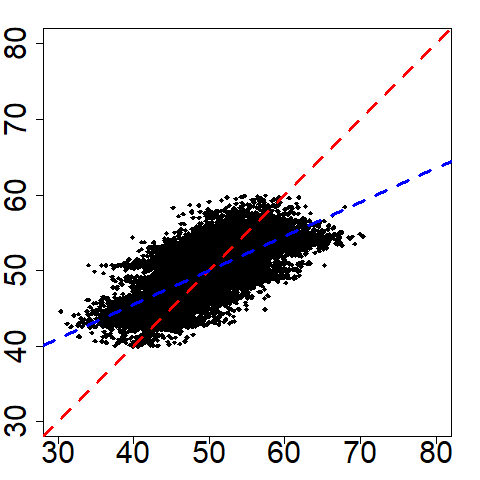 | | 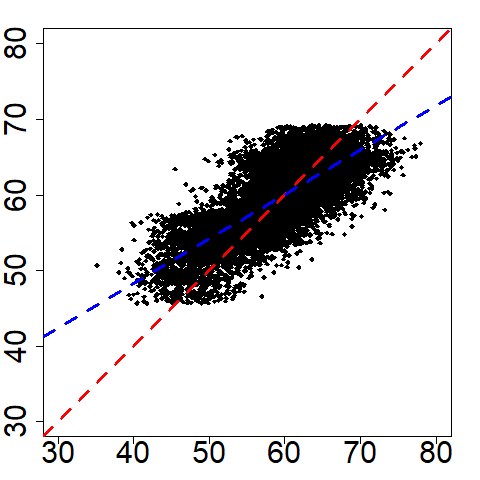 | | 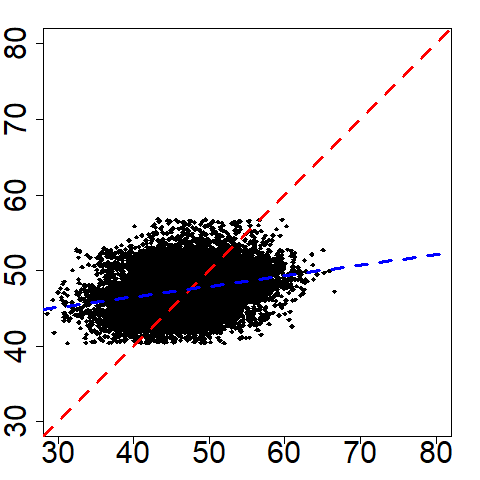 | | 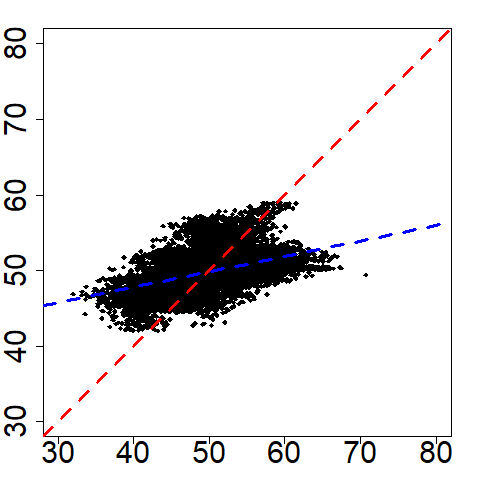 | | 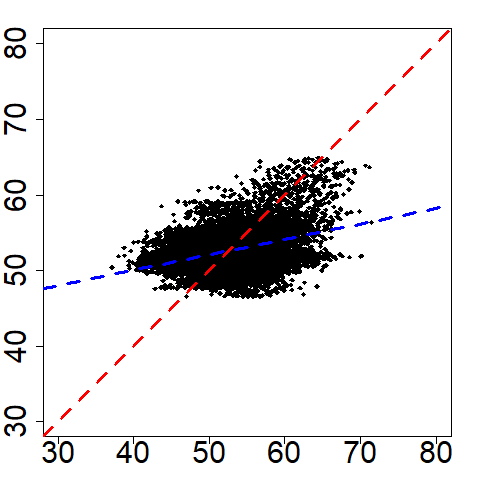 | | 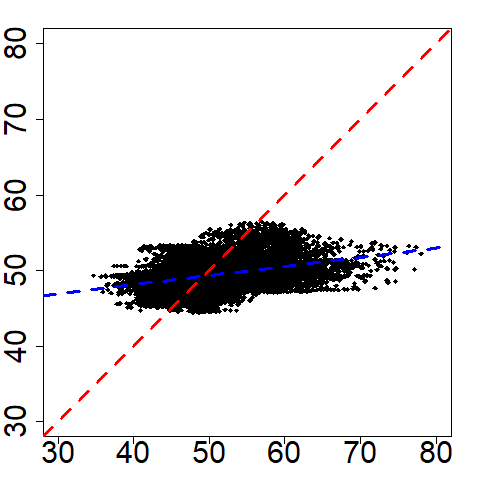 | | 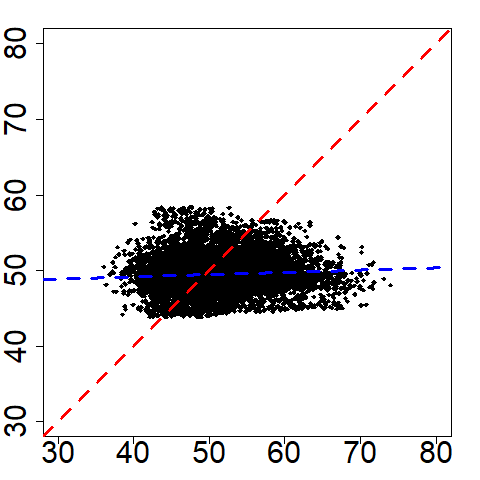 |
| LUR | | 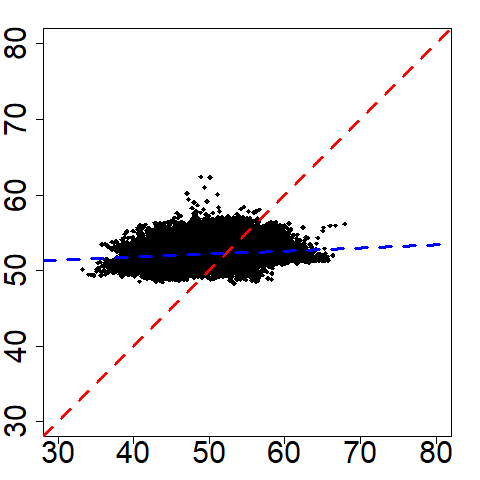 | | 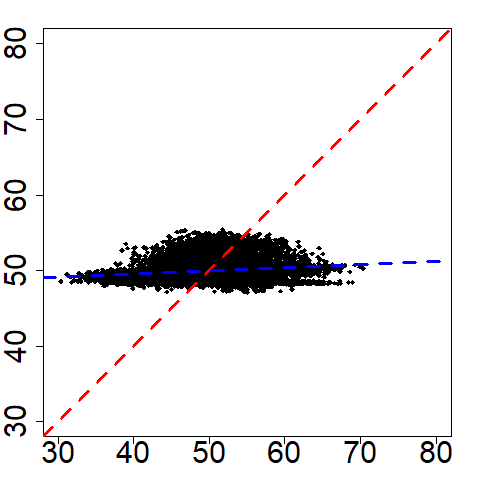 | | 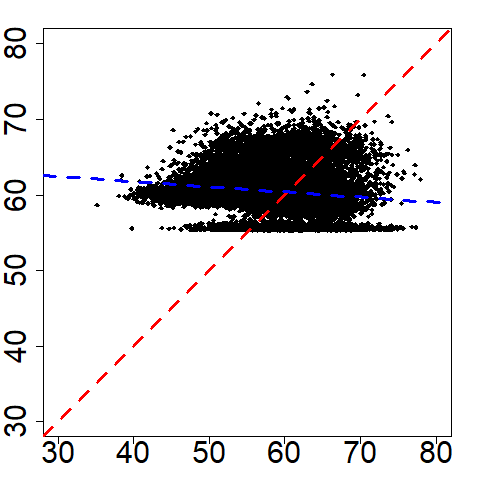 | | 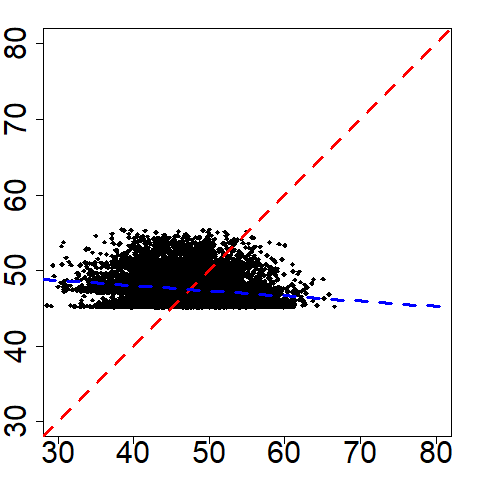 | | 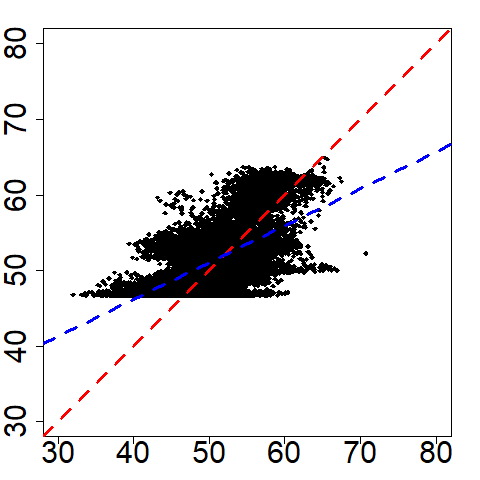 | | 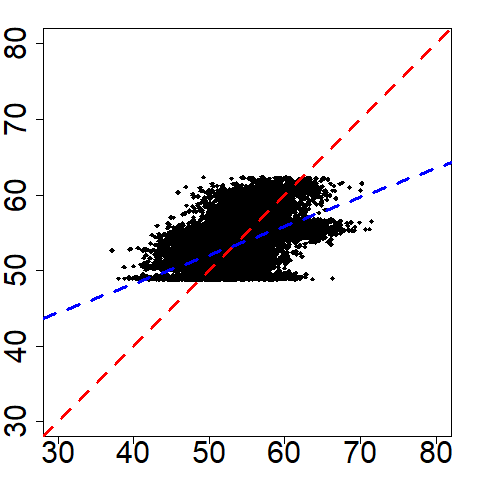 | | 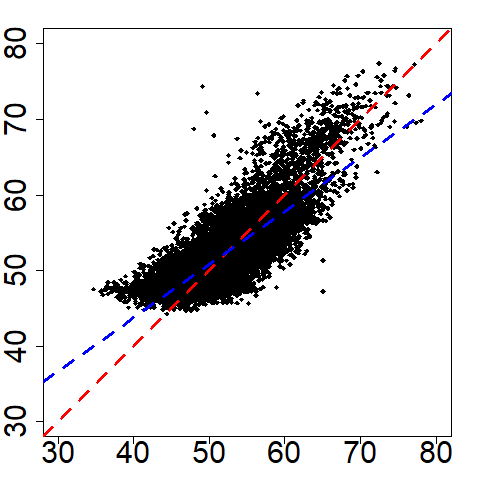 | | 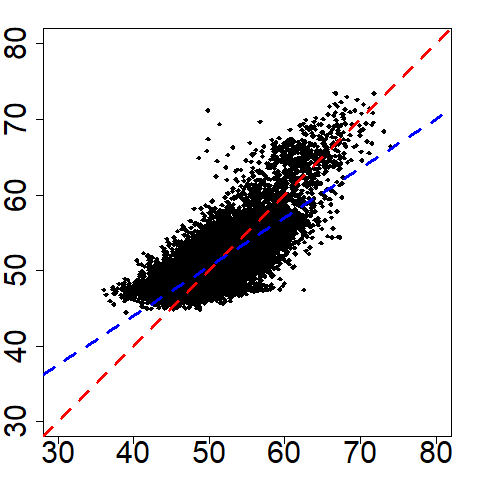 |
| UK | | 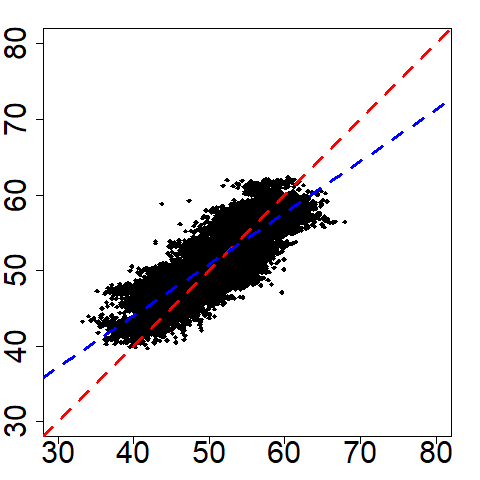 | | 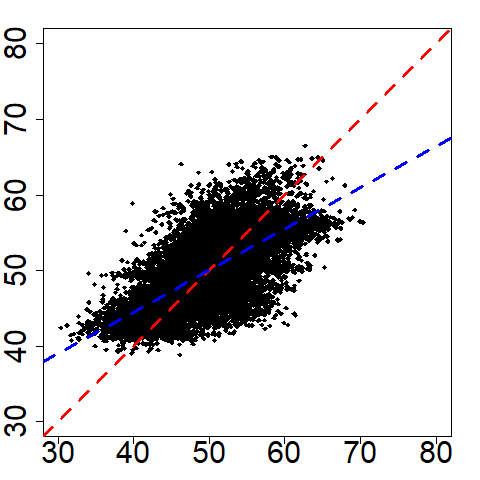 | | 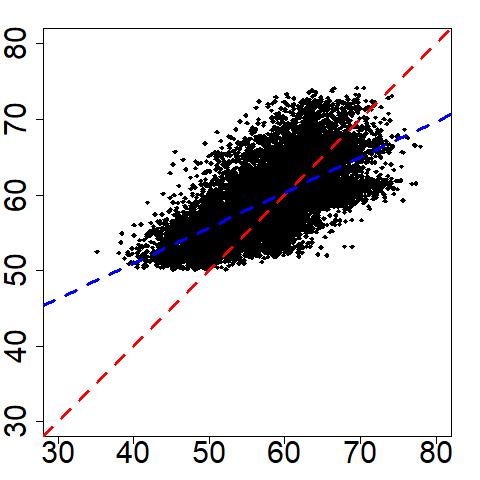 | | 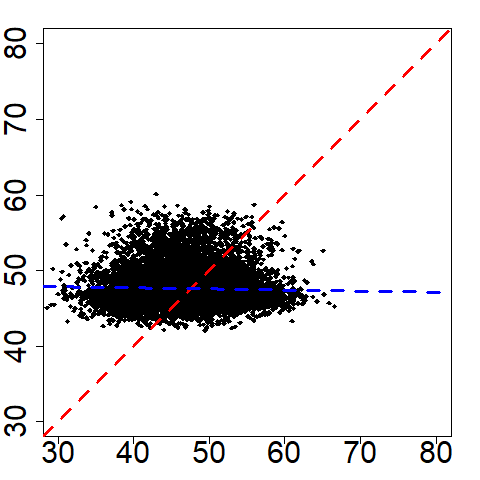 | | 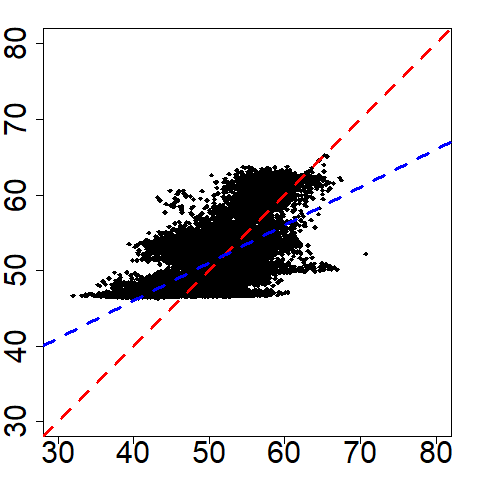 | | 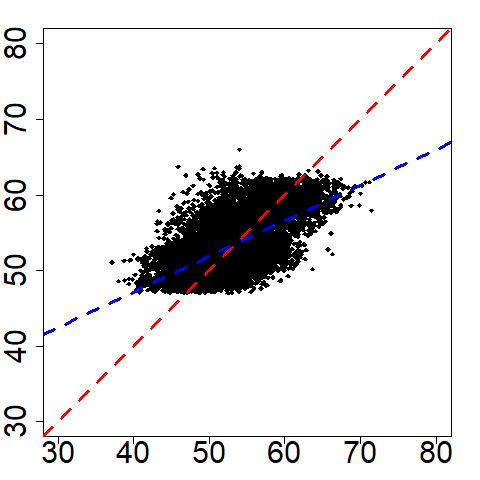 | | 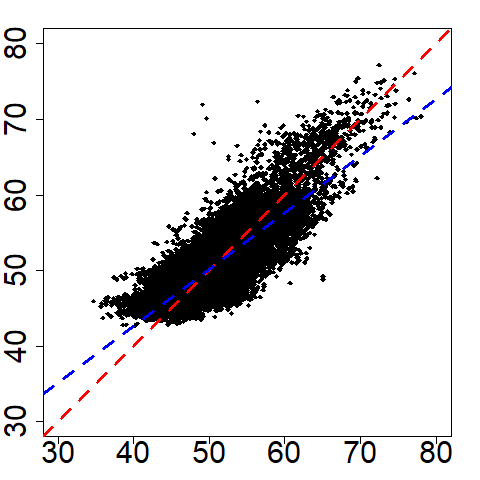 | | 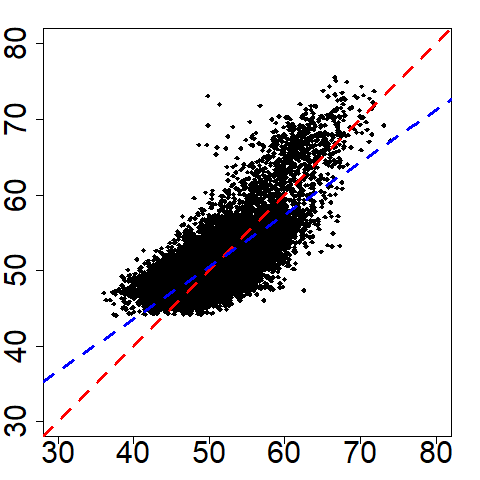 |
| AA | | 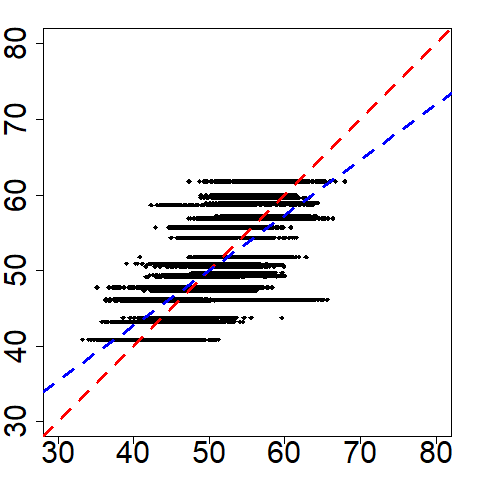 | | 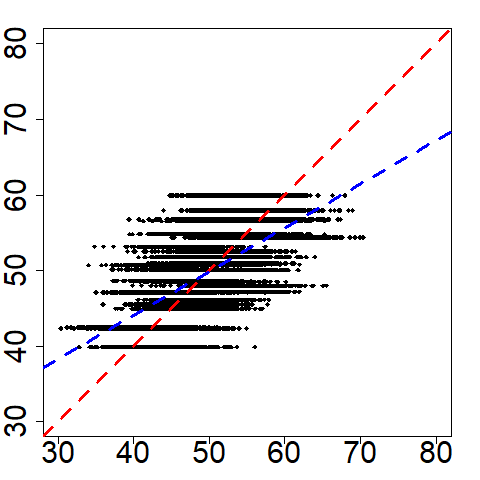 | | 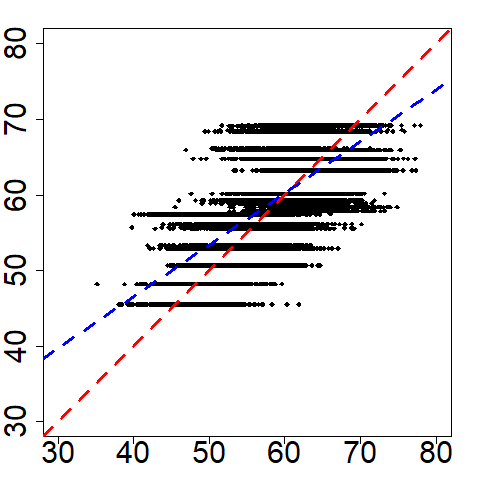 | | 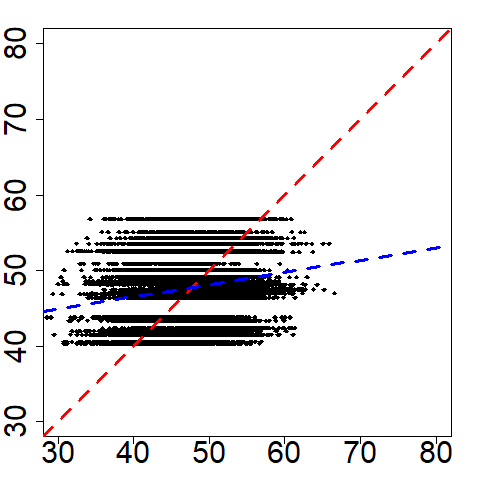 | | 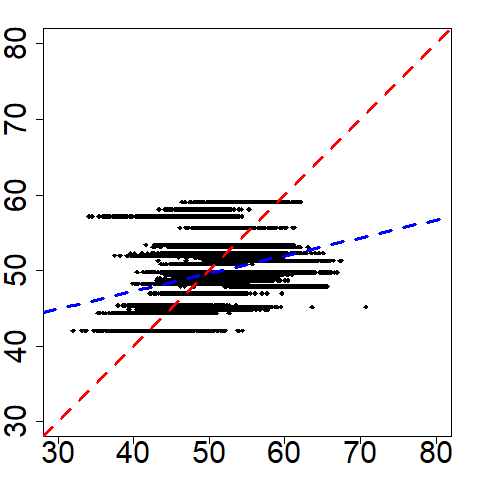 | | 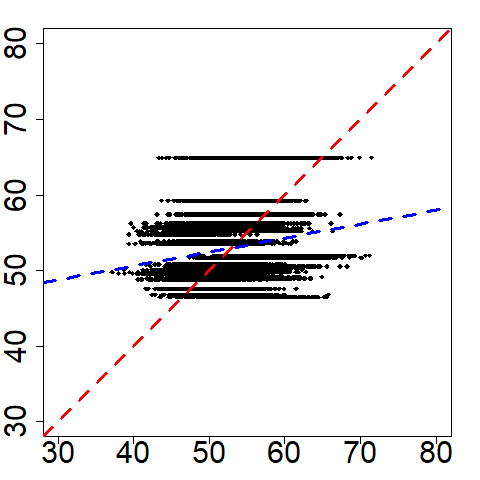 | | 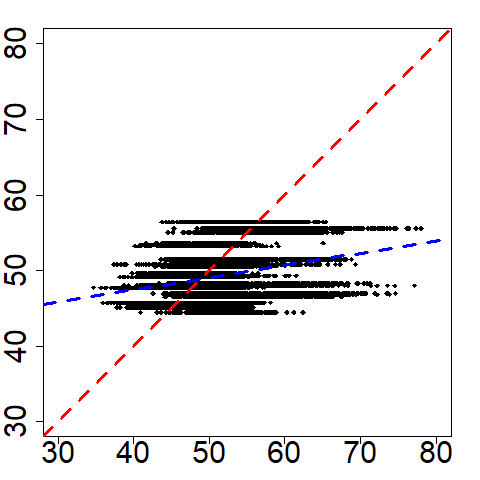 | | 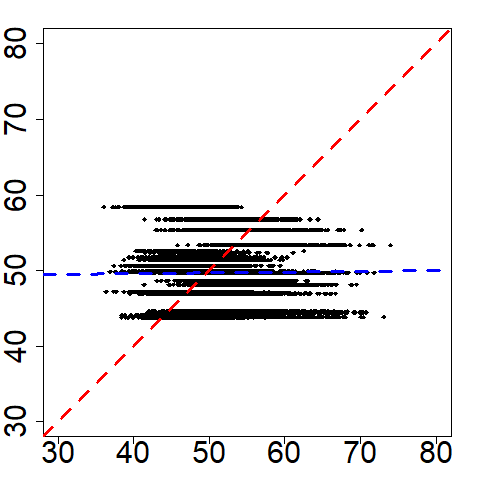 |
| UKD | | 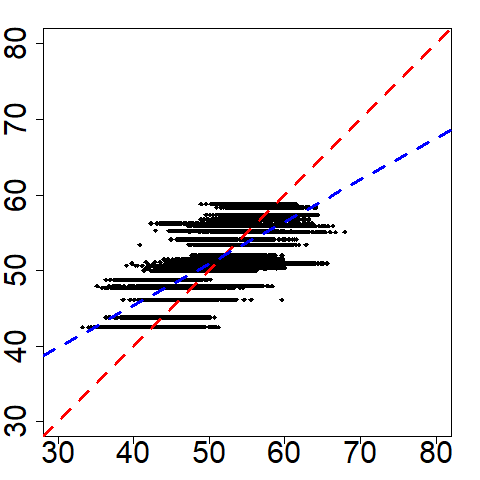 | | 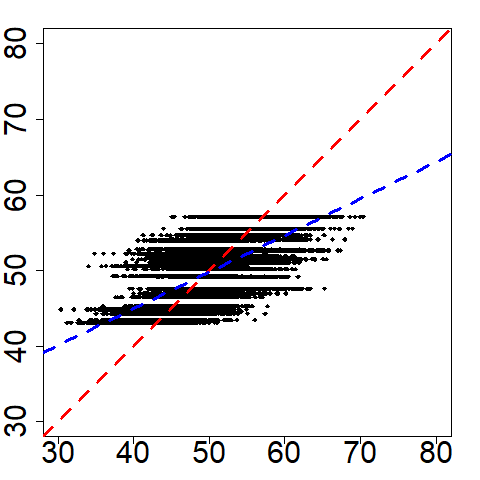 | | 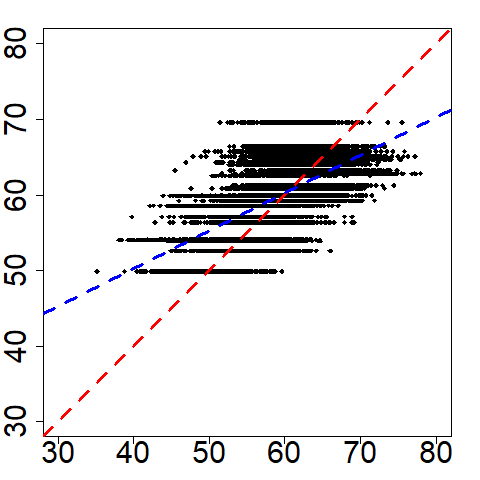 | | 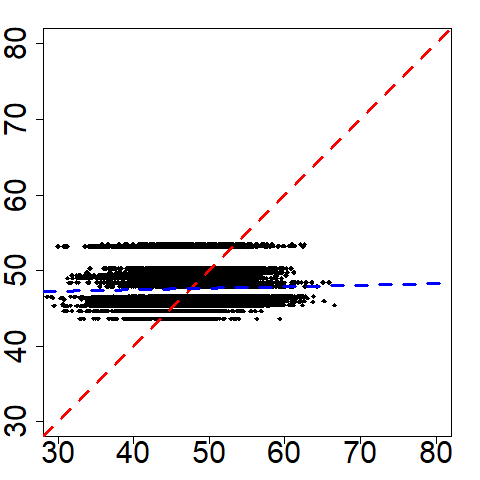 | | 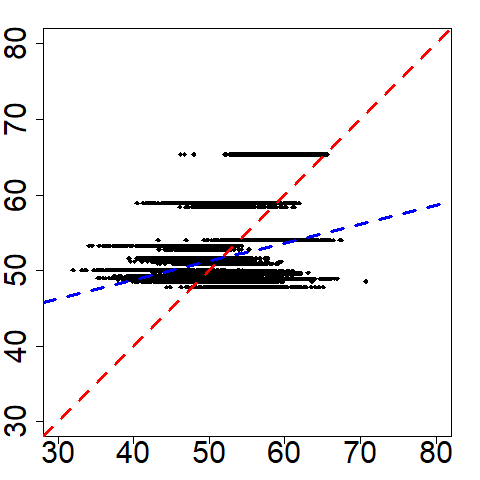 | | 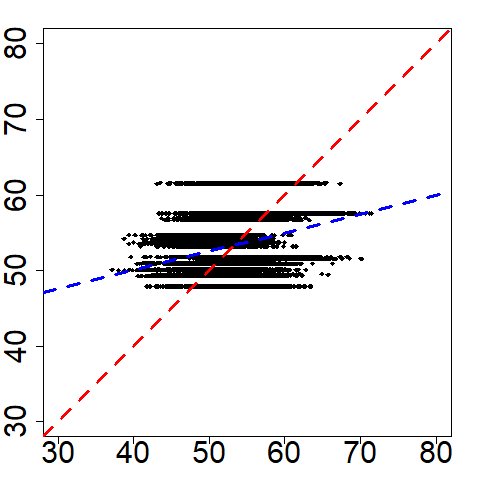 | | 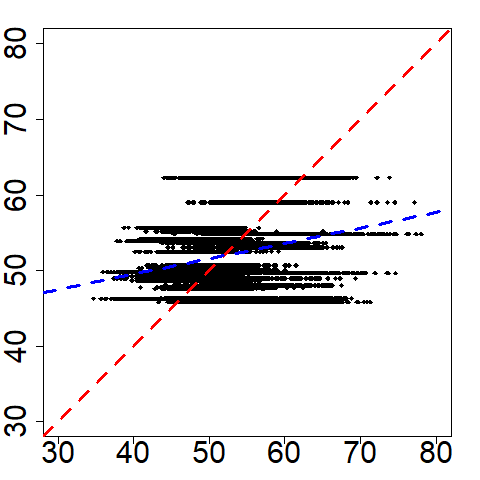 | | 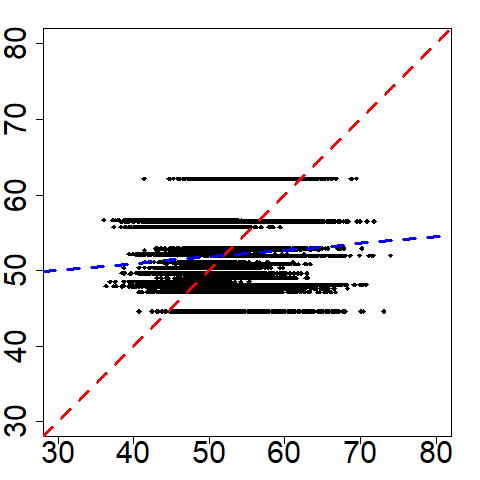 |
| UKNA | | 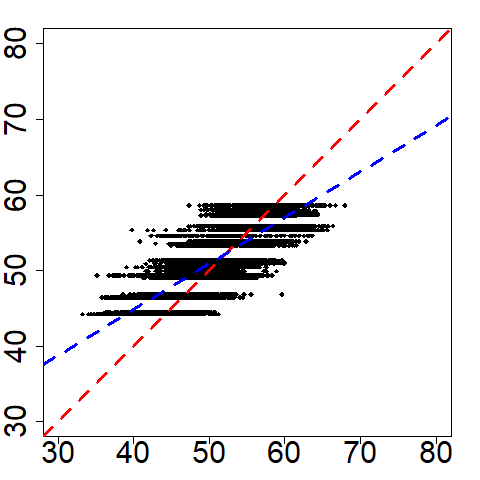 | | 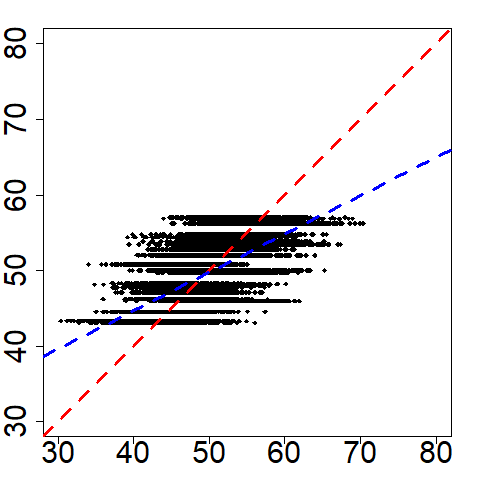 | | 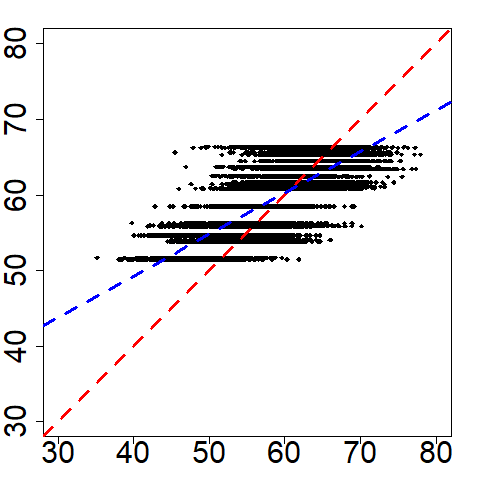 | | 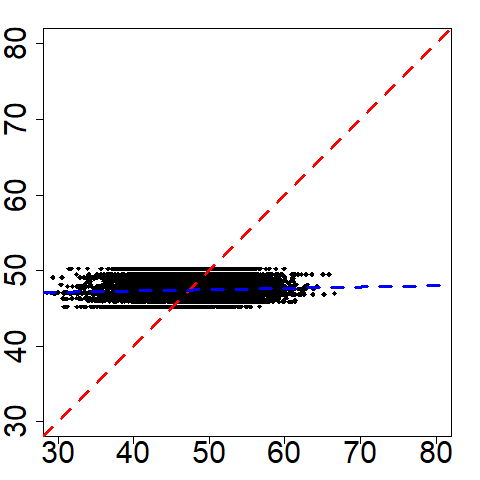 | | 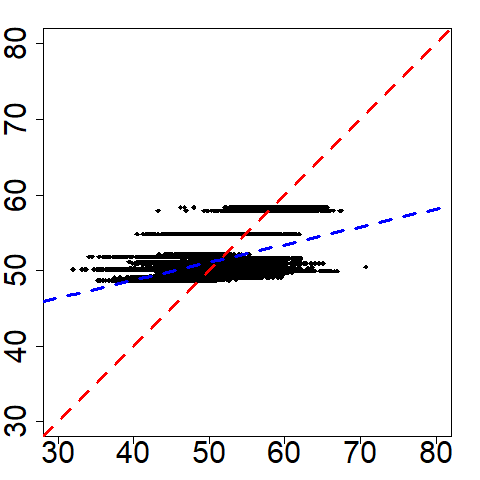 | | 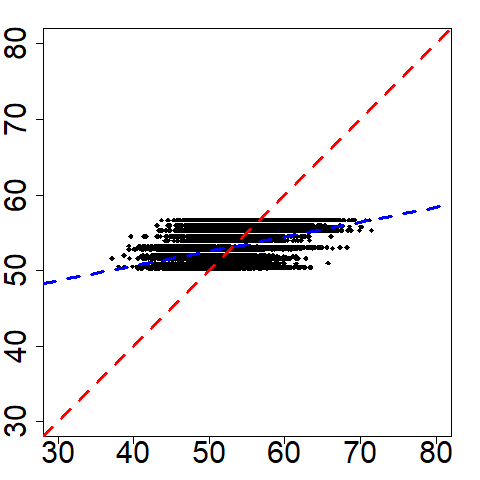 | | 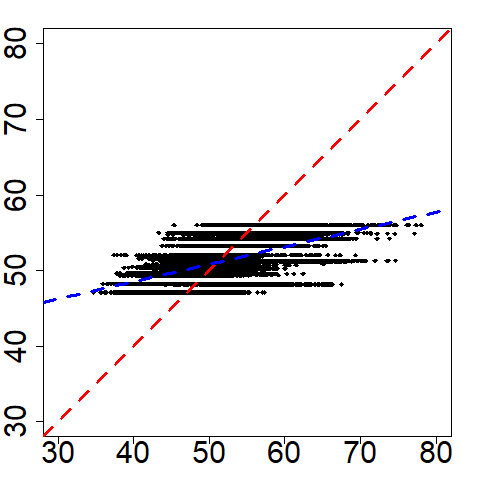 | | 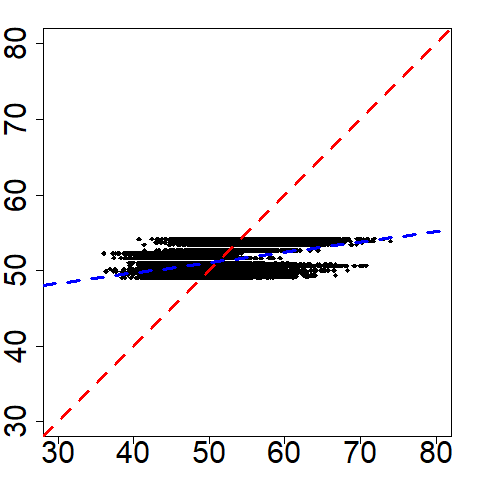 |
| UKCA | | 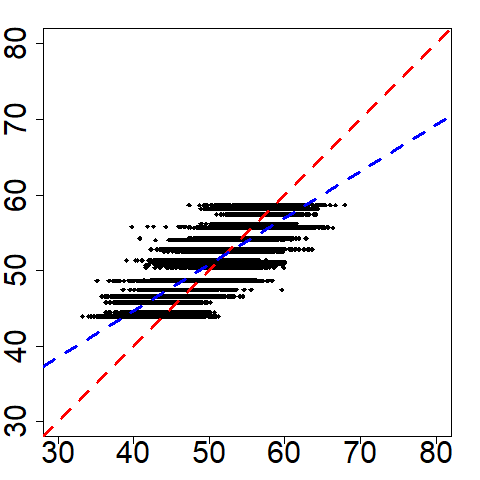 | | 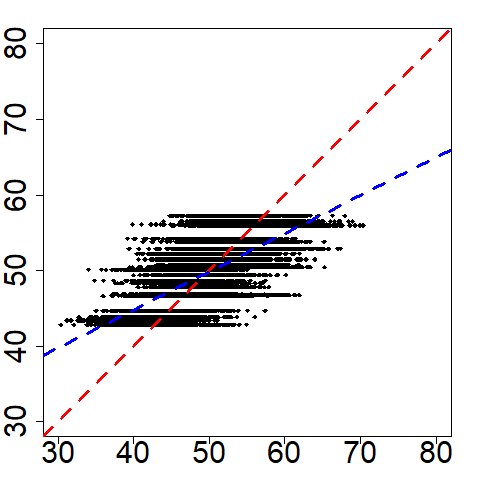 | | 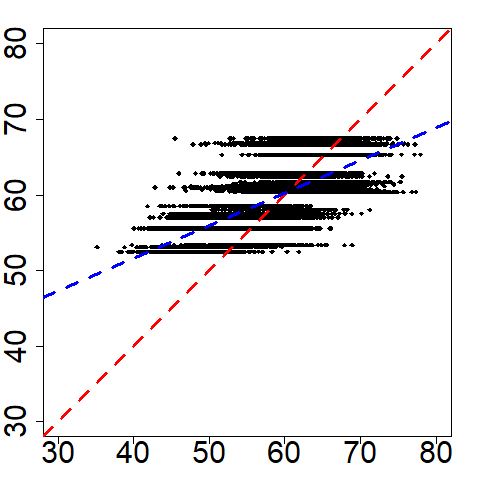 | | 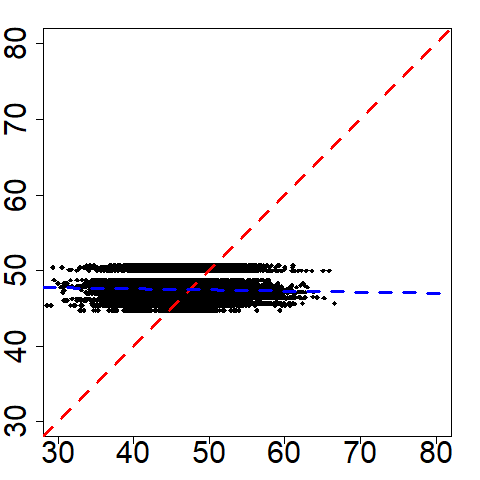 | | 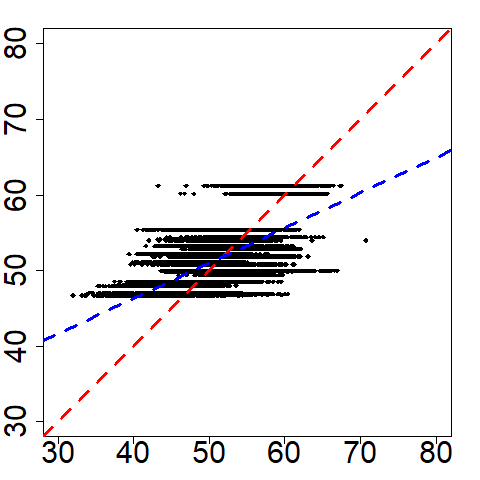 | | 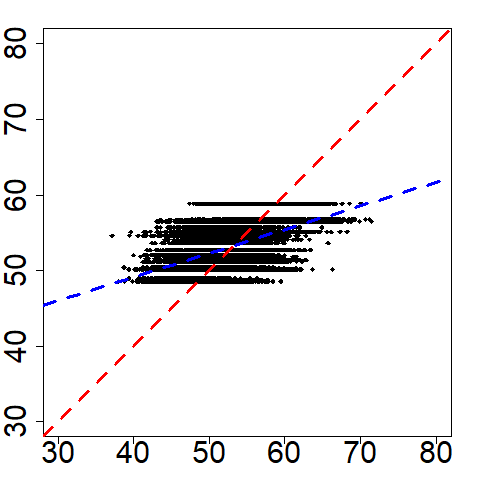 | | 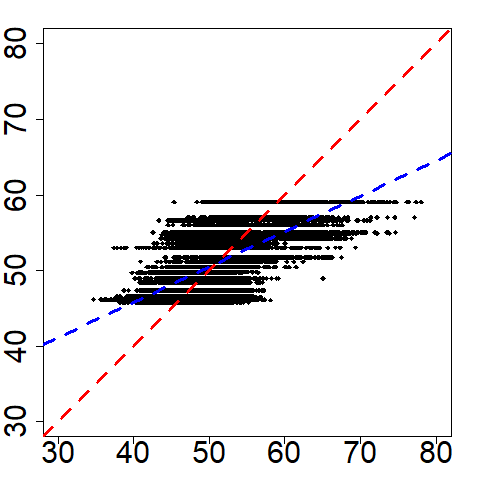 | | 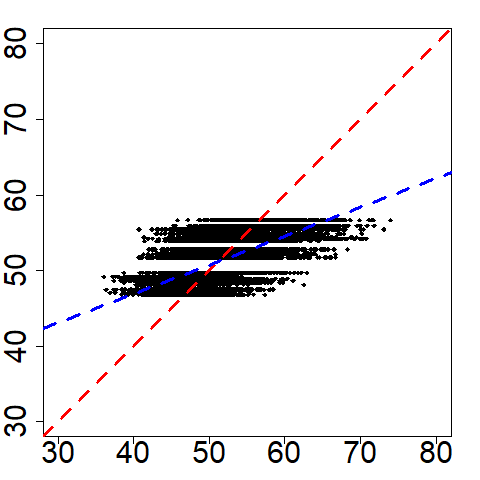 |
| UKGA | | 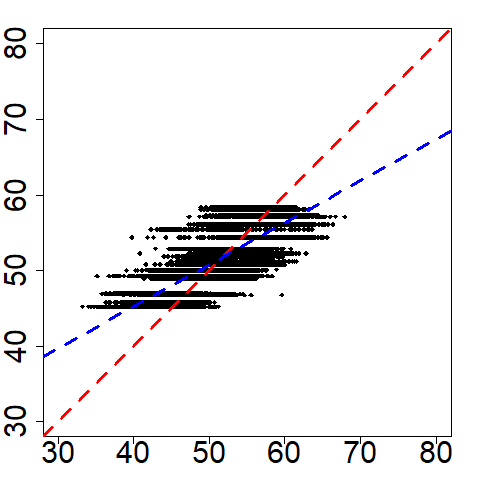 | | 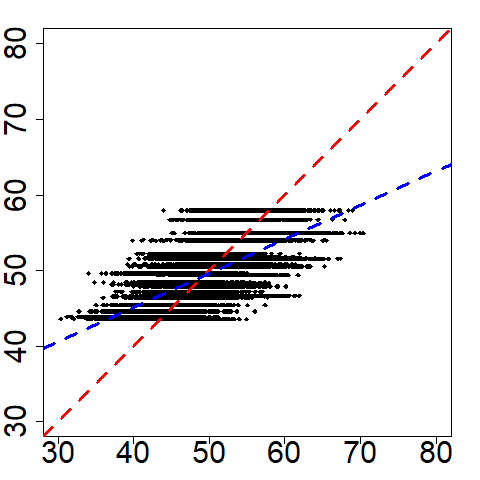 | | 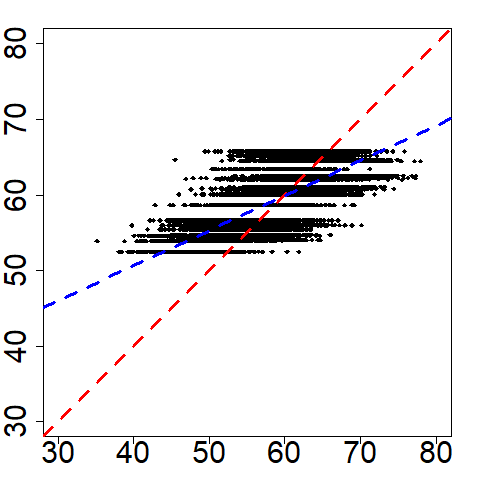 | | 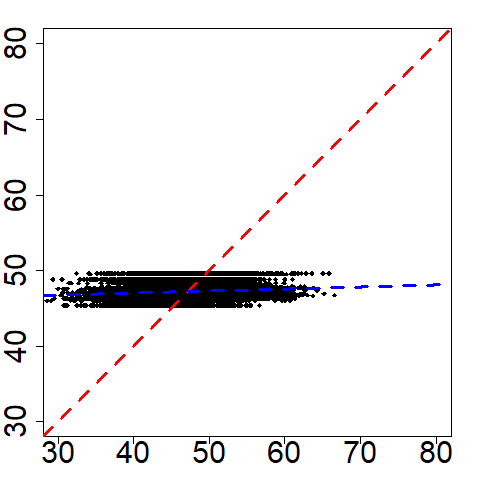 | | 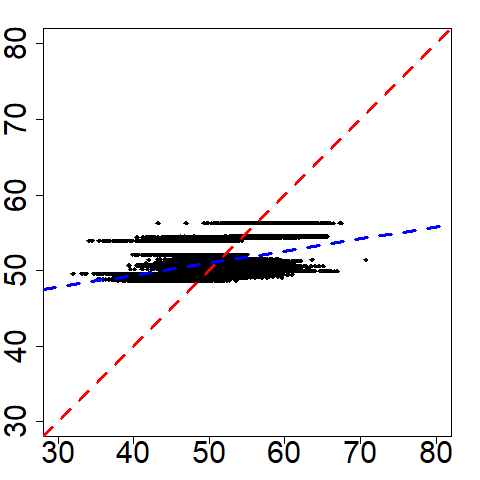 | | 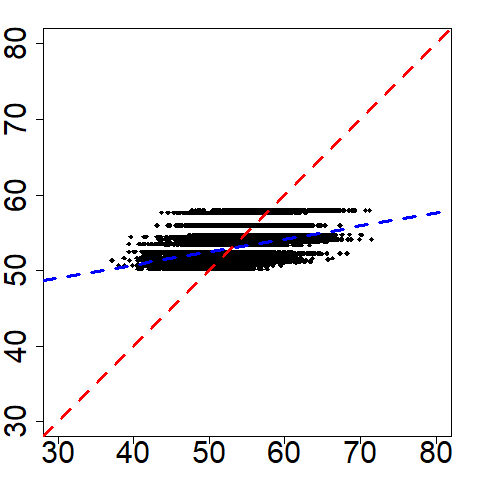 | | 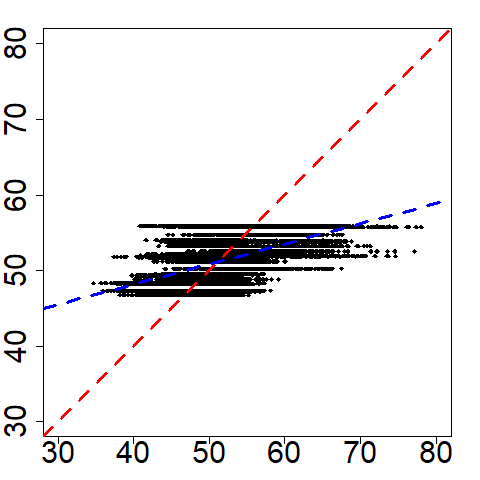 | | 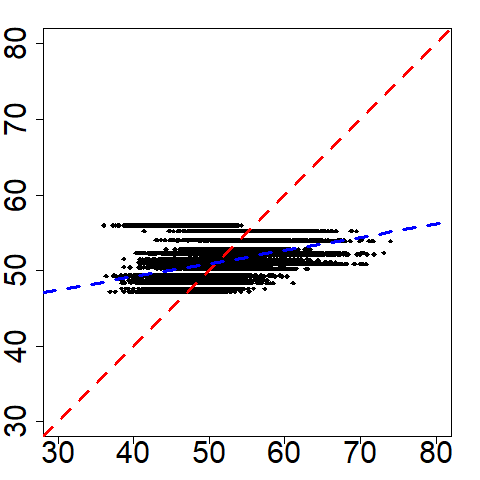 |

**Figure S5.** Bar plots of bias (multiplied by 100) of low birth weight effect estimates for true and nine types of predicted PM_10_ concentrations (TE: true exposure, NM: nearest monitor, IDWA: inverse distance weight average, LUR: Land-use regression, AA: area average, UK: universal kriging, UKD: UK prediction at governmental offices; UKNA: district average of UK predictions at 422 neighbourhood community centers; UKCA: district average of UK predictions at 16,230 census tract centroids; UKGA: district average of UK predictions at 610 1-km grid coordinates) depending on the address data availability (yellow and orange bars for complete and incomplete addresses, respectively) by eight environmental scenarios

| 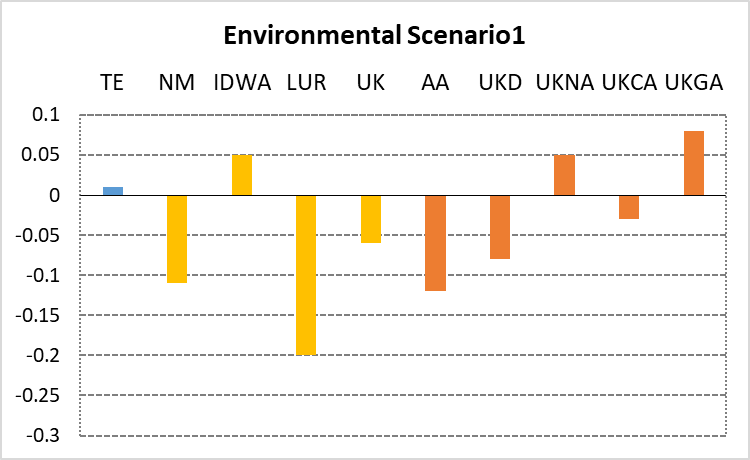  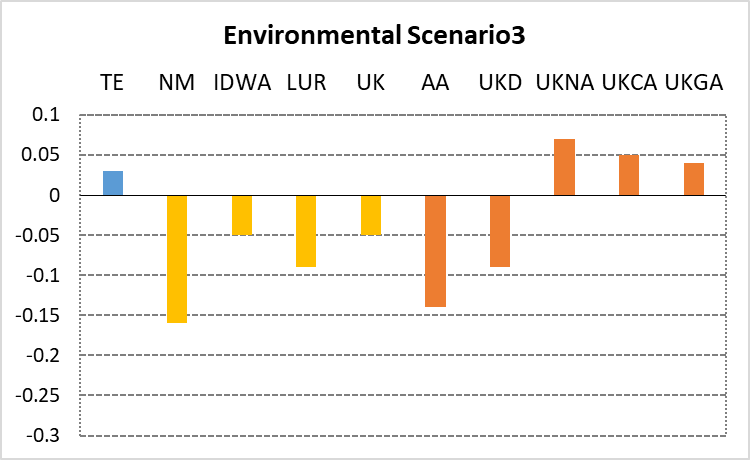 | 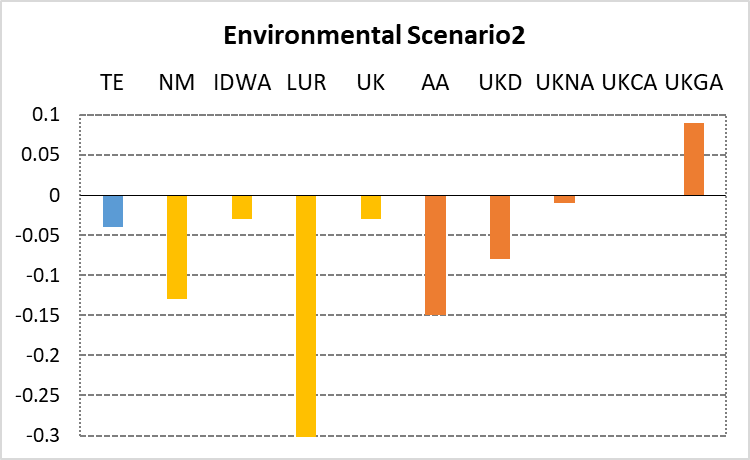  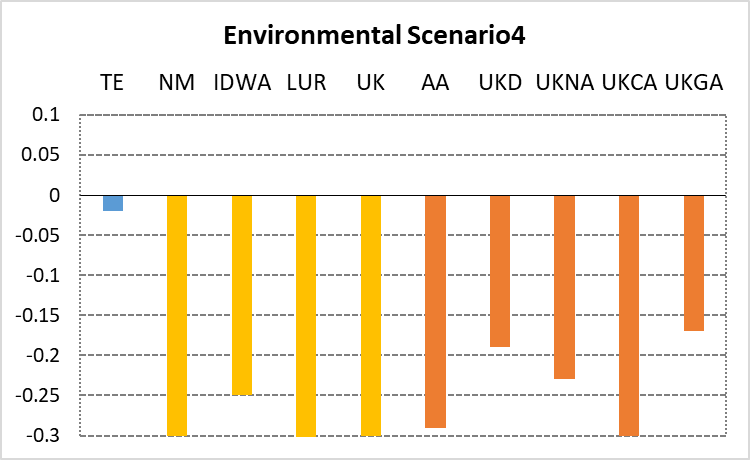 |
| --- | --- |
| 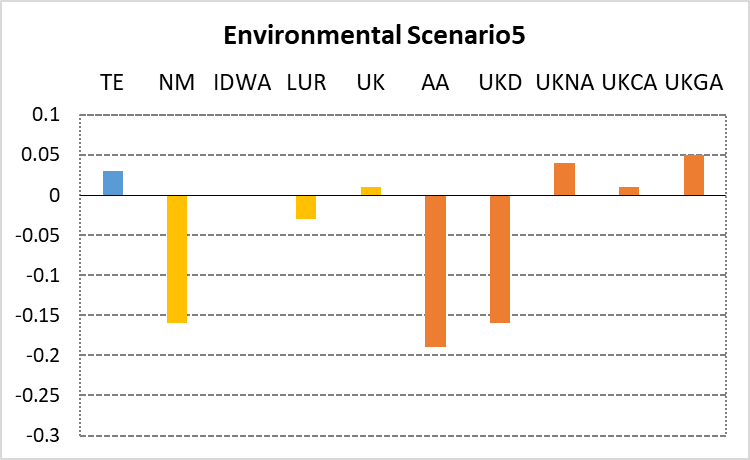 | 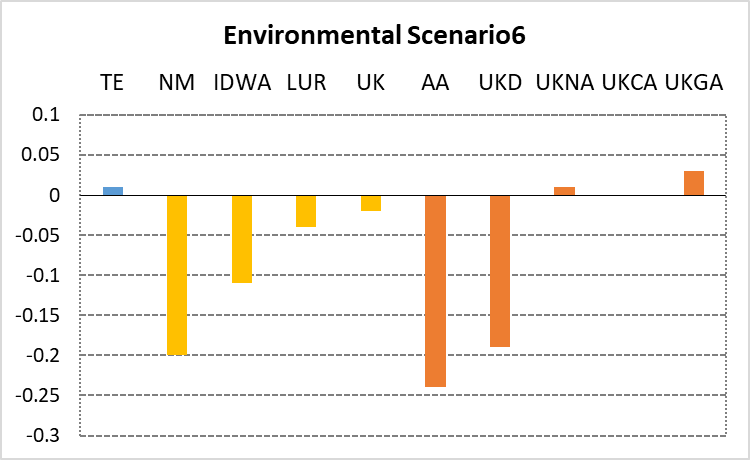 |
| 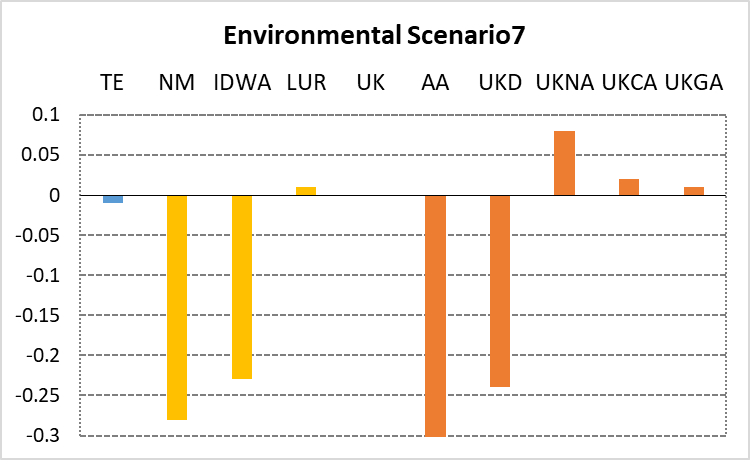 | 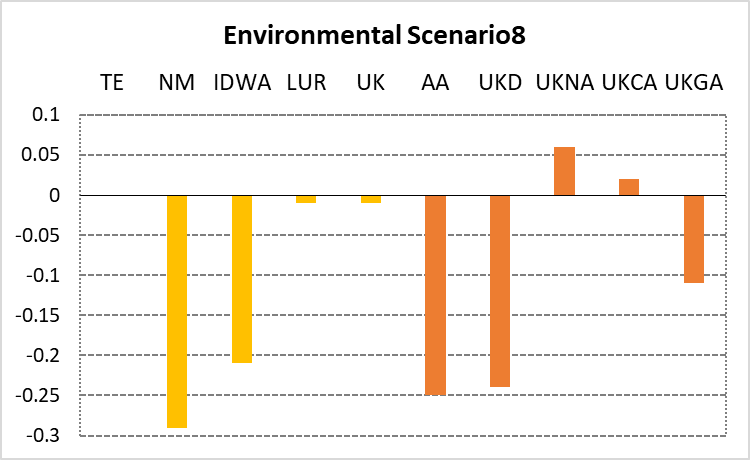 |
|  |  |

**FigureS6.** Scatter plots for logarithm of health effect estimates using true (x-axis) and predicted (y-axis) annual-average PM_10_ concentrations at home addresses of 46,007 mothers over 1,000 simulations across major environmental scenario (ES2, ES3, ES5, ES8). Blue, red, black solid, and black dotted lines are for best-fitted, 45-degree lines, the true coefficient value, and the average of the fitted coefficient values, respectively.

| Prediction Method | ES2 | ES3 | ES5 | ES8 |
| --- | --- | --- | --- | --- |
| NM | 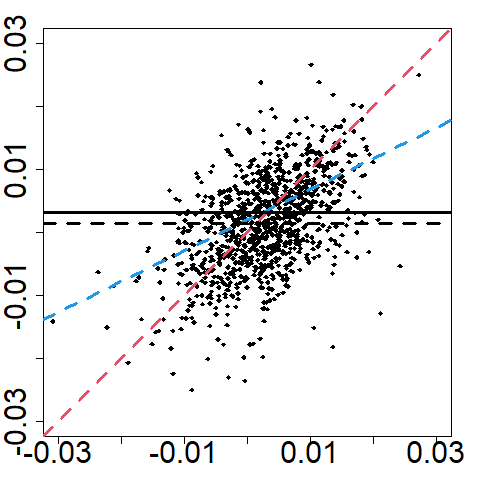 | 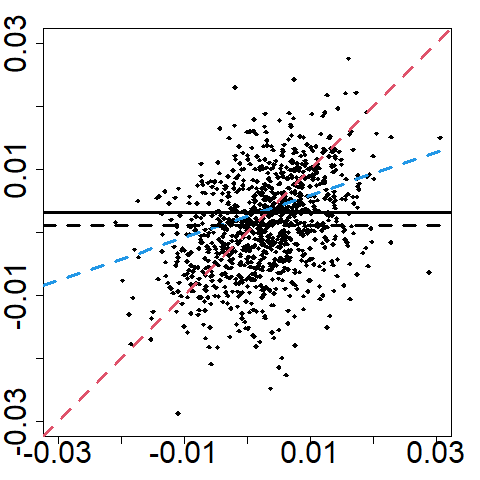 | 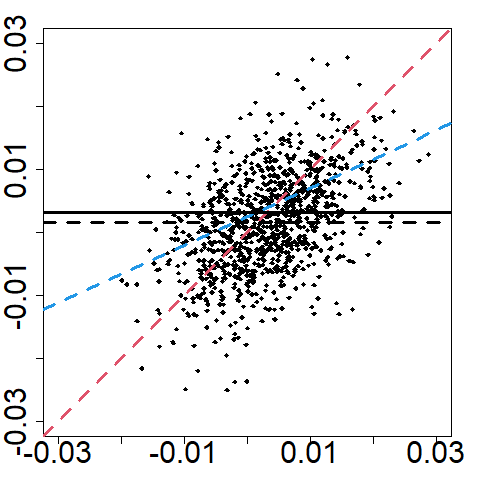 | 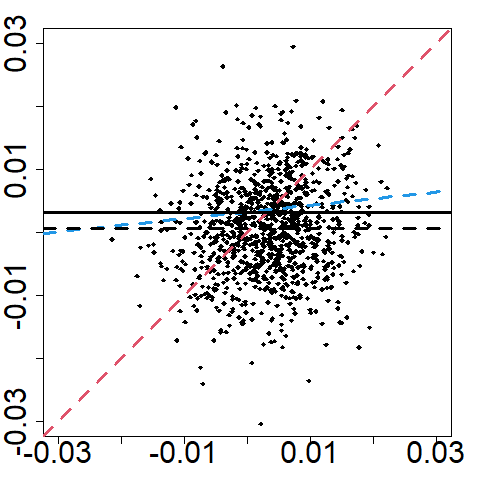 |
| IDWA | 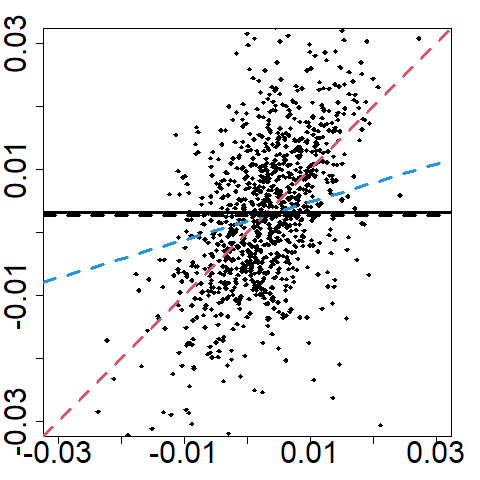 | 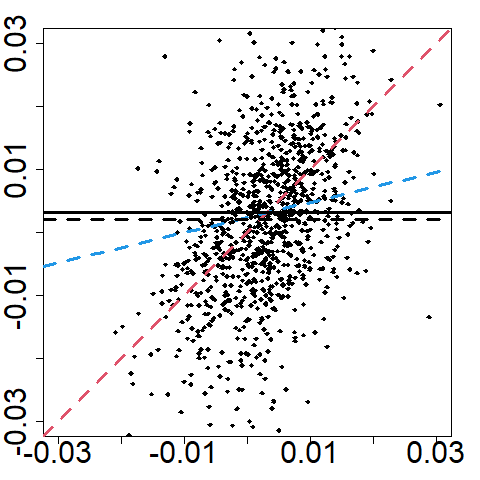 | 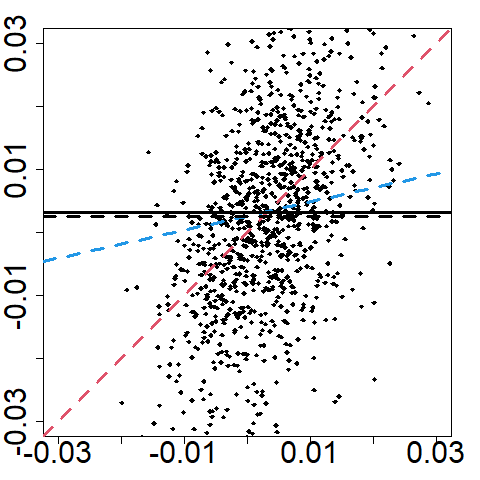 | 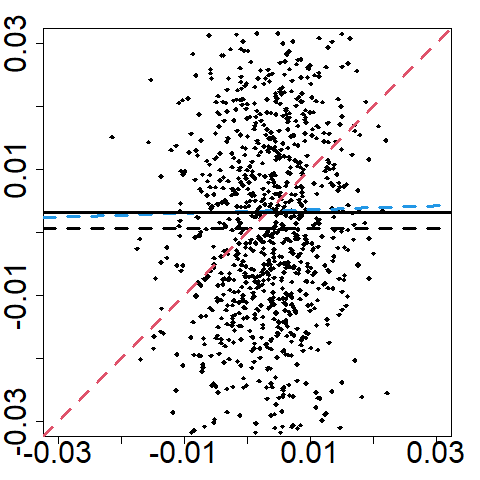 |
| LUR | 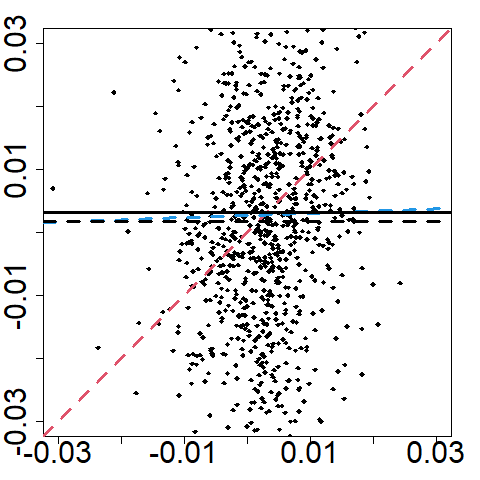 | 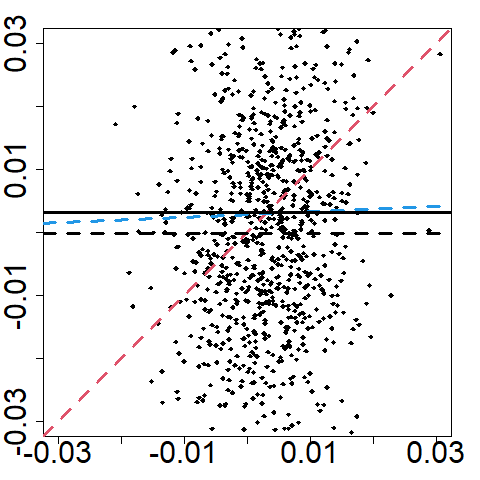 |  |  |
| UK |  |  |  |  |
| AA |  |  |  |  |
| UKD |  |  |  |  |
| UKNA |  |  |  |  |
| UKCA |  |  |  |  |
| UKGA |  |  |  |  |

**Figure S7.** Bar plots of root mean square error (filled bar), average standard error (empty bar), and coverage probability (grey point) of low birth weight effect estimates for true and nine types of predicted PM_10_ concentrations depending on the address data availability (yellow and orange bars for complete and incomplete addresses, respectively) by eight environmental scenarios.

|  |  |
| --- | --- |
|  |  |
|  |  |
|  |  |

**Figure S8.** True-positive rate for low birth weight effect estimates of nine types of predicted PM_10_ concentrations depending on the address data availability (yellow and orange bars for complete and incomplete addresses, respectively) by eight environmental scenarios

|  |  |
| --- | --- |
|  |  |
|  |  |
|  |  |
|  |  |

**Table S1.** Exposure assessment approaches of nine exposure prediction methods in our simulation study

| Prediction method | Assessment of individual exposure | Address Availability |
| --- | --- | --- |
| Nearest monitor (NM) | PM concentration measured at the nearest regulatory monitoring site to mother’s home address | Complete |
| Inverse distance weighted average (IDWA) | Average concentration across regulatory monitoring sites weighted by inverse squared Euclidean distance from mother’s home | Complete |
| Land use regression (LUR) | Concentration at mother’s home predicted based on a regression model including geographic predictors^a^ | Complete |
| Universal kriging (UK) | Concentration at mother’s home predicted by a geostatistical method including geographic predictors and spatial correlation | Complete |
| Area averaging (AA) | Concentration averaged across all monitoring sties in a district | Incomplete |
| Universal kriging - District (UKD) | Concentration predicted at a district governmental office by UK | Incomplete |
| Universal kriging - Neighborhood Average (UKNA) | District-specific concentration averaged over UK predictions at neighborhood community centers | Incomplete |
| Universal kriging - Census Track Average (UKCA) | District-specific concentration averaged over UK predictions at census tract centroids | Incomplete |
| Universal kriging - Grid Average (UKGA) | District-specific concentration averaged over UK predictions at 1-km grid coordinates | Incomplete |

^a^ Length of major road b (100m buffer); Proportion of water surface land use (500m); Number of construction companies (1000m); Distance to the nearest bus stop; Number of employees in construction industries (100m)

**Table S2.** Mean and standard deviation of true and predicted PM_10_ annual average concentrations at home addresses of 46,007 mothers by address availability, eight environmental scenarios (ES1-ES8), and exposure prediction methods in the 1^st^ simulation.

|  |  | Complete address | | | | Incomplete address | | | | |
| --- | --- | --- | --- | --- | --- | --- | --- | --- | --- | --- |
|  | TE^b^ | NM^b^ | IDWA^b^ | LUR^b^ | UK^b^ | AA^b^ | UKD^b^ | UKNA^b^ | UKCA^b^ | UKGA^b^ |
| ES1 | 51.16(5.56) ^a^ | 50.94(5.73) | 51.42(5.47) | 51.14(4.16) | 52.27(1.56) | 51.59(4.24) | 51.63(4.53) | 51.61(4.24) | 51.6(4.27) | 51.49(3.97) |
| ES2 | 49.80(5.97) | 49.75(5.34) | 49.83(5.20) | 49.94(3.80) | 49.97(1.59) | 49.71(4.13) | 49.91(4.80) | 49.68(4.49) | 49.81(4.38) | 49.49(4.04) |
| ES3 | 58.55(6.45) | 59.23(6.45) | 59.27(5.89) | 59.27(4.88) | 60.52(3.43) | 59.56(4.56) | 59.66(4.46) | 59.45(4.63) | 59.60(4.09) | 59.21(4.02) |
| ES4 | 46.90(5.11) | 47.68(4.59) | 47.43(3.81) | 47.48(2.71) | 47.52(1.99) | 47.61(2.56) | 47.57(2.29) | 47.42(1.21) | 47.52(1.50) | 47.25(1.04) |
| ES5 | 50.91(5.26) | 49.87(4.18) | 50.47(5.33) | 49.98(2.35) | 51.51(4.30) | 51.46(4.16) | 51.51(4.33) | 51.26(2.65) | 51.41(4.07) | 51.19(2.13) |
| ES6 | 53.05(4.66) | 53.02(4.57) | 53.22(4.68) | 52.67(2.62) | 53.21(3.45) | 53.32(4.08) | 53.37(3.54) | 53.19(2.07) | 53.22(2.86) | 52.99(2.00) |
| ES7 | 51.61(5.81) | 49.34(3.51) | 50.51(4.86) | 49.49(1.89) | 51.97(5.09) | 51.89(4.43) | 51.42(5.33) | 51.22(2.48) | 51.24(4.16) | 51.28(2.98) |
| ES8 | 51.19(5.17) | 49.66(4.00) | 50.53(4.58) | 49.53(2.03) | 51.35(4.32) | 51.97(4.29) | 51.34(4.64) | 51.21(1.74) | 51.19(3.14) | 51.13(2.41) |

^a^ Mean (Standard deviation)

^b^ TE: true exposure; UK: universal kriging_;_ AA: area-average UKD: UK prediction at governmental offices without aggregation; UKNA: district average based on UK predictions at 422 neighbourhood community centers; UKCA: district average of UK predictions at 16,230 census tract centroids; UKGA: district average of UK predictions at 610 1-km grid coordinates

**Table S3.** Pearson correlation coefficients and regression coefficients between true and predicted annual-average PM_10_ concentrations at home addresses of 46,007 mothers in the 1st simulation

|  |  | ES1 |  |  | ES2 |  |  | ES3 |  |  | ES4 |  |  |
| --- | --- | --- | --- | --- | --- | --- | --- | --- | --- | --- | --- | --- | --- |
| Address | Exposure prediction ^d^ | R^a^ | Inter ^b^ | Slope ^c^ | R | Inter | Slope | R | Inter | Slope | R | Inter | Slope |
| Complete | NM  IDWA  LUR  UK | 0.62  0.62  0.02  0.70 | 11.69  21.01  50.28  16.78 | 0.78  0.59  0.04  0.68 | 0.49  0.50  0.03  0.47 | 19.52  27.46  47.87  22.56 | 0.61  0.45  0.04  0.55 | 0.52  0.61  0.02  0.46 | 20.83  24.77  64.50  32.29 | 0.66  0.59  -0.07  0.47 | 0.04  0.07  0.03  0.00 | 40.80  40.82  50.73  48.26 | 0.14  0.14  -0.07  -0.02 |
| Incomplete | AA  UKD  UKNA  UKCA  UKGA | 0.50  0.52  0.64  0.64  0.60 | 13.51  23.33  20.47  20.15  23.14 | 0.73  0.55  0.61  0.62  0.55 | 0.42  0.49  0.45  0.47  0.44 | 20.91  25.55  24.46  24.70  27.05 | 0.58  0.49  0.51  0.50  0.45 | 0.47  0.50  0.59  0.47  0.55 | 19.18  30.26  27.30  34.30  32.11 | 0.68  0.50  0.55  0.43  0.46 | 0.03  0.00  0.01  0.00  0.02 | 40.10  46.73  46.57  48.23  46.01 | 0.16  0.02  0.02  -0.02  0.03 |
|  |  | ES5 |  |  | ES6 |  |  | ES7 |  |  | ES8 |  |  |
| Address | Exposure prediction | R | Inter | Slope | R | Inter | Slope | R | Inter | Slope | R | Inter | Slope |
| Complete | NM  IDWA  LUR  UK | 0.30  0.21  0.36  0.37 | 22.39  39.61  26.58  25.99 | 0.55  0.20  0.49  0.50 | 0.18  0.13  0.27  0.38 | 30.84  41.87  32.96  28.44 | 0.42  0.20  0.38  0.47 | 0.13  0.14  0.65  0.67 | 34.92  43.30  15.57  12.66 | 0.30  0.12  0.71  0.75 | 0.03  0.01  0.60  0.59 | 42.52  48.08  18.11  16.05 | 0.16  0.03  0.65  0.69 |
| Incomplete | AA  UKD  UKNA  UKCA  UKGA | 0.09  0.10  0.22  0.36  0.15 | 37.86  38.95  39.23  27.73  43.12 | 0.24  0.25  0.24  0.47  0.16 | 0.04  0.08  0.19  0.26  0.16 | 43.30  40.24  42.95  36.68  43.79 | 0.18  0.25  0.19  0.31  0.17 | 0.07  0.07  0.29  0.43  0.27 | 41.07  41.41  39.42  27.09  37.59 | 0.16  0.20  0.23  0.47  0.27 | 0.00  0.01  0.17  0.40  0.14 | 49.13  47.43  44.12  31.62  42.13 | 0.01  0.09  0.14  0.38  0.18 |

^a^ Pearson correlation coefficient between true and predicted annual-average PM_10_;^b^ OLS regression intercept coefficient; ^c^ OLS regression slope coefficient

^d^ TE: true exposure; UK: universal kriging; AA: area-average UKD: UK prediction at governmental offices without aggregation; UKNA: district average based on UK predictions at 422 neighbourhood community centers; UKCA: district average of UK predictions at 16,230 census tract centroids; UKGA: district average of UK predictions at 610 1-km grid coordinates

**Table S4.** Properties of effect estimates of true (TE) and predicted PM_10_ annual average concentrations on low birth weight over 1,000 simulations by address availability, nine exposure prediction methods (NM, IDWA, LUR, UK, AA, UKD, UKNA, UKCA, and UKGA), and four environmental scenarios without mean structure (ES1-ES4)

|  |  | ES1 |  |  |  | ES2 |  |  |  | ES3 |  |  |  | ES4 |  |  |  |
| --- | --- | --- | --- | --- | --- | --- | --- | --- | --- | --- | --- | --- | --- | --- | --- | --- | --- |
| Address | Exposure  prediction | Bias^a^ | RMSE^b^ | ASE^c^ | CP^d^ | Bias | RMSE | ASE | CP | Bias | RMSE | ASE | CP | Bias | RMSE | ASE | CP |
|  | TE | 0.01 | 1.51 | 0.77 | 0.96 | -0.04 | 1.48 | 0.78 | 0.95 | 0.03 | 1.48 | 0.75 | 0.95 | -0.02 | 1.43 | 0.74 | 0.95 |
| Complete | UK | -0.11  0.05  -0.20  -0.06 | 1.56  2.54  4.34  2.19 | 0.78  1.35  2.66  1.19 | 0.96  0.96  0.94  0.96 | -0.13  -0.03  -0.33  -0.03 | 1.54  2.50  4.51  2.41 | 0.83  1.35  2.54  1.45 | 0.93  0.95  0.96  0.94 | -0.16  -0.05  -0.09  -0.05 | 1.54  2.54  4.65  2.72 | 0.82  1.32  2.63  1.58 | 0.94  0.95  0.96  0.94 | -0.30  -0.25  -0.37  -0.30 | 1.50  2.65  4.54  3.47 | 0.76  1.36  2.65  2.05 | 0.92  0.96  0.93  0.94 |
| Incomplete | AA  UKD  UKNA  UKCA  UKGA | -0.12  -0.08  0.05  -0.03  0.08 | 1.57  2.19  2.74  2.53  3.17 | 0.82  1.17  1.48  1.40  1.77 | 0.95  0.95  0.96  0.96  0.96 | -0.15  -0.08  -0.01  0.00  0.09 | 1.55  2.42  3.27  2.84  3.83 | 0.83  1.41  1.95  1.74  2.38 | 0.94  0.94  0.94  0.94  0.94 | -0.14  -0.09  0.07  0.05  0.04 | 1.56  2.75  4.08  3.32  4.83 | 0.80  1.62  2.67  2.00  3.63 | 0.95  0.94  0.94  0.95  0.94 | -0.29  -0.19  -0.23  -0.30  -0.17 | 1.51  3.58  6.47  4.54  7.92 | 0.79  1.91  3.85  2.96  4.53 | 0.94  0.96  0.94  0.92  0.95 |

^a^ Bias multiplied by 100

^b^ Root mean square error multiplied by 100

^c^ Average standard error multiplied by 100

^d^ Coverage probability of 95% confidence interval

^e^ TE: true exposure; UK: universal kriging; AA: area-average UKD: UK prediction at governmental offices without aggregation; UKNA: district average based on UK predictions at 422 neighbourhood community centers; UKCA: district average of UK predictions at 16,230 census tract centroids; UKGA: district average of UK predictions at 610 1-km grid coordinates

**Table S5.** Properties of effect estimates of true (TE) and predicted PM_10_ annual average concentrations on low birth weight over 1,000 simulations by address availability, nine exposure prediction methods (NM, IDWA, LUR, UK, AA, UKD, UKNA, UKCA, UKGA), and four environmental scenarios without mean structure (ES5-ES8)

|  |  | ES5 |  |  |  | ES6 |  |  |  | ES7 |  |  |  | ES8 |  |  |  |
| --- | --- | --- | --- | --- | --- | --- | --- | --- | --- | --- | --- | --- | --- | --- | --- | --- | --- |
| Address | Exposure prediction | Bias^a^ | RMSE^b^ | ASE^c^ | CP^d^ | Bias | RMSE | ASE | CP | Bias | RMSE | ASE | CP | Bias | RMSE | ASE | CP |
|  | TE | 0.03 | 1.50 | 0.78 | 0.95 | 0.01 | 1.49 | 0.75 | 0.95 | -0.01 | 1.40 | 0.71 | 0.95 | 0.00 | 1.40 | 0.71 | 0.95 |
| Complete | UK | -0.16  0.00  -0.03  0.01 | 1.53  3.08  2.42  2.06 | 0.77  1.65  1.31  1.10 | 0.96  0.95  0.95  0.95 | -0.20  -0.11  -0.04  -0.02 | 1.52  3.03  2.45  2.11 | 0.76  1.54  1.29  1.10 | 0.95  0.95  0.95  0.96 | -0.28  -0.23  0.01  0.00 | 1.53  3.60  1.75  1.70 | 0.80  1.90  0.94  0.89 | 0.94  0.94  0.94  0.93 | -0.29  -0.21  -0.01  -0.01 | 1.54  3.69  1.76  1.73 | 0.79  1.90  0.89  0.88 | 0.94  0.94  0.96  0.96 |
| Incomplete | AA  UKD  UKNA  UKCA  UKGA | -0.19  -0.16  0.04  0.01  0.05 | 1.76  2.02  3.10  2.33  3.71 | 0.95  1.07  1.68  1.26  2.04 | 0.95  0.94  0.94  0.94  0.95 | -0.24  -0.19  0.01  0.00  0.03 | 1.76  2.07  3.23  2.38  3.83 | 0.89  1.12  1.80  1.23  2.09 | 0.96  0.95  0.95  0.95  0.95 | -0.31  -0.24  0.08  0.02  0.01 | 2.00  1.89  3.89  2.20  3.24 | 1.03  0.99  2.07  1.19  1.70 | 0.94  0.94  0.94  0.94  0.95 | -0.25  -0.24  0.06  0.02  -0.11 | 2.04  1.93  4.07  2.23  3.31 | 1.05  1.03  2.14  1.16  1.73 | 0.95  0.95  0.94  0.96  0.95 |

^a^ Bias multiplied by 100

^b^ Root mean square error multiplied by 100

^c^ Average standard error multiplied by 100

^d^ Coverage probability of 95% confidence interval

^e^ TE: true exposure; UK: universal kriging; AA: area-average UKD: UK prediction at governmental offices without aggregation; UKNA: district average based on UK predictions at 422 neighbourhood community centers; UKCA: district average of UK predictions at 16,230 census tract centroids; UKGA: district average of UK predictions at 610 1-km grid coordinates
